# The impact of early-life rearing conditions on the porcine gut microbiota and immune system

**DOI:** 10.1186/s42523-025-00492-y

**Published:** 2025-12-05

**Authors:** Luke Comer, Muhammad Zeeshan Akram, Haoran Zhao, Peiyang Huo, Ester Arévalo Sureda, Chuanpi Xiao, Hikmat Ullah Khan Usman, Pawel Siegien, José Wavreille, Jan Aerts, Nadia Everaert

**Affiliations:** 1 Nutrition & Animal Microbiota EcoSystems Lab, Department of Biosystems, KU Leuven, Heverlee, 3001 Belgium; 2 Augmented Intelligence for Data Analytics Lab, Department of Biosystems, KU Leuven, Heverlee, 3001 Belgium; 3 Precision Livestock and Nutrition Laboratory, TERRA Teaching and Research Centre, Gembloux Agro-Bio Tech, University of Liège, Gembloux, 5030 Belgium; 4 Centre Wallon de Recherches Agronomiques, Gembloux, 5030 Belgium; 5 BioNanotechnology Institute, School of Food Engineering, Ludong University, Yantai, Shandong China

**Keywords:** Pig microbiota, Gut physiology, Immunology, Spleen, Early-life microbiota

## Abstract

**Background:**

Early life represents an unparalleled window in the life of the pig in which the gut microbiota interacts with its host’s naïve immune system. Yet, modern swine production often favours conditions that promote production efficiency rather than enriched microbiota development, the long-term consequences of which remain poorly understood. This study sought to analyse the long-term impacts of early-life rearing conditions on the gut microbiota until day 90, and in turn, its physiological and immunological consequences. We established two rearing conditions from farrowing until day 90: enriched, microbiota-enhancing husbandry characterised by weaning at 6 weeks and the provision of litter material throughout; and restricted, microbiota-depleting husbandry comprising weaning at 3 weeks and antibiotic administration from days 2 to 9. The day 42 faecal, and day 90 ileal and faecal microbiotas underwent 16 S V1-V9 rRNA gene sequencing. Intestinal and faecal volatile fatty acids were measured via gas chromatography, haematological parameters were assessed from whole blood, and serum immunoglobulin G was measured. Immune-focused gene expression in the spleen and ileum was also measured via qPCR.

**Results:**

The faecal microbiota exhibited differential β-diversity by group at both timepoints. On day 90, enriched pigs exhibited significantly elevated ileal villus height to crypt depth ratios, which were negatively correlated with serum IgG. Conversely, restricted pigs had more branched-chain fatty acids in the colon and faeces, alongside signs of heightened immune activity, with haematology showing enhanced neutrophil activation, and elevated lymphocyte and IgG levels. In the spleen, gene sets comprising genes for the pro-inflammatory cytokines IL-6, IL-15 and IFN-γ were upregulated among restricted pigs, while enriched pigs exhibited better-primed innate immune systems.

**Conclusions:**

These findings demonstrate long-term impacts of early-life rearing on faecal microbiota composition. We furthermore observed a potential shift towards inflammation and altered haematology associated with the microbiota.

**Supplementary Information:**

The online version contains supplementary material available at 10.1186/s42523-025-00492-y.

## Graphical Abstract

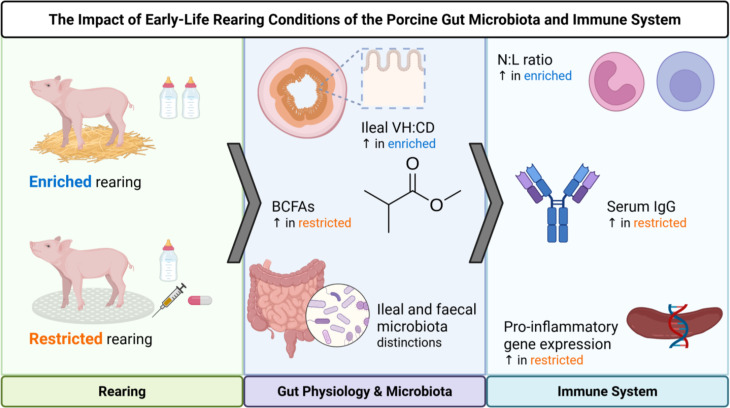

## Background

The gut microbiota has drawn considerable interest in recent years given the profound interplay between its constituent microorganisms and its host. Of particular interest is the early-life period, wherein microbiota-immune interactions prove particularly influential in shaping later immune capacity [1, 2]. The pig is no exception to this, with microbiota interventions having gained traction in the swine industry. While much progress has been made in understanding the importance of the microbiota in shaping host health, information regarding the longer-term, physiological consequences of real-world conditions remains scarce.

In favour of efficiency in the commercial sector, farmers must often make compromises which deviate from the conditions in which the pig and its microbiota co-evolved. Firstly, weaning in commercial farming is often performed when piglets are just three weeks of age. Considering the relative prematurity of the piglet’s gut at this age, weaning acts as a source of physiological stress when a plant-based diet, rich in complex carbohydrates is encountered [3, 4], notwithstanding the additional psychological stress. As such, the faecal microbiota displays significant changes such as decreased *Lactobacillus* and reduced diversity of other commensal bacteria in the short term [5–8]. This places the piglet at risk of microbial dysbiosis, and conditions such as post-weaning diarrhoea [9]. Secondly, while the use of growth-promoting and prophylactic antibiotics is today banned in much of the world, antibiotics remain a frontline defence in cases of disease. Antibiotic use is well known to result in taxonomic and diversity-related disruptions [10–12], although the exact effects vary greatly depending on the dose and intestinal site studied. Such antibiotic-induced disruptions can render the gut vulnerable to pathobionts or opportunistic pathogens. Lastly, environmental factors such as the provision of bedding material can differ greatly in commercial farming. Straw and other litter materials have been shown to alter the microbiota’s taxonomic composition, although the exact nature and duration of these effects have proven inconsistent across studies [13–15].

While the aforementioned factors have been shown to convey impacts on the gut microbiota, and in turn, the health of its host, there exists much uncertainty regarding their exact nature and longevity. Besides, previous studies differ in what they evaluate, and the precise effects, integrating the microbiota and more systemic physiology and immunology remain less understood.

The present study sought to replicate two rearing conditions representing two extreme ends of the farming spectrum, hereafter referred to as “restricted” and “enriched”. Having established these rearing conditions, the study aimed to determine their impacts on the composition of the gut microbiota, and in turn, analysed the subsequent impacts on host physiology and immunology. We hypothesised that pigs raised in the enriched system would have faecal microbiotas distinct from their restricted counterparts, while the restricted pigs would exhibit greater markers of ileal and systemic inflammation at the end of the experiment at 90 days of age.

## Materials and methods

### Animal ethics statement

This animal experiment was carried out at the Centre Wallon de Recherches Agronomiques (CRA-W) (Gembloux, Belgium). Ethical approval was obtained from the ethical committee at the University of Liège (ULiège) with the file number 22-2523, with recognition by the KU Leuven ethical committee.

### Animal husbandry and experimental design

A total of four litters of 16 Piétrain × Belgian Landrace piglets were selected at farrowing (day 0) and assigned evenly to the enriched and restricted groups (two litters per group). Dams were controlled for parity, with each experimental group consisting of one parity six and one parity five sow.

Following farrowing, all piglets received iron injections, and were vaccinated against coccidiosis (Baycox^®^). All male piglets were castrated on day 3.

Straw was removed from the restricted piglets’ pens shortly after farrowing. From days 2 to 9, the restricted group underwent broad-spectrum antibiotic treatments to cause early-life microbiota disruption. A double treatment was chosen in order to maximise possible microbiota perturbation compared to a single antibiotic. This consisted of trimethoprim-sulfamethoxazole, a synergistic folate pathway inhibitor [16], and amoxicillin, a broad-spectrum β-lactam, both frequently used for porcine gastrointestinal infections [17]. Firstly, piglets received an intramuscular injection of trimethoprim-sulfamethoxazole from days 2 to 4 at a dosage of 30 mg/kg bodyweight. Thereafter, amoxicillin was administered orally from days 5 to 9, at a dose of 20 mg/kg bodyweight. Piglets were kept in the same pen within their respective groups, and enriched pigs were provided with wood shavings as bedding material from weaning onwards. At weaning, the two litters of each group were added to the same pen, with both groups remaining in the same barn, but in separate pens.

Diet was the same for both groups, the nutritional composition of which can be found in Table S1. Solid feed was provided from day 14 while all piglets were still suckling. For the restricted group, weaning took place on day 21 while enriched piglets were weaned on day 42.

**Fig. 1:**
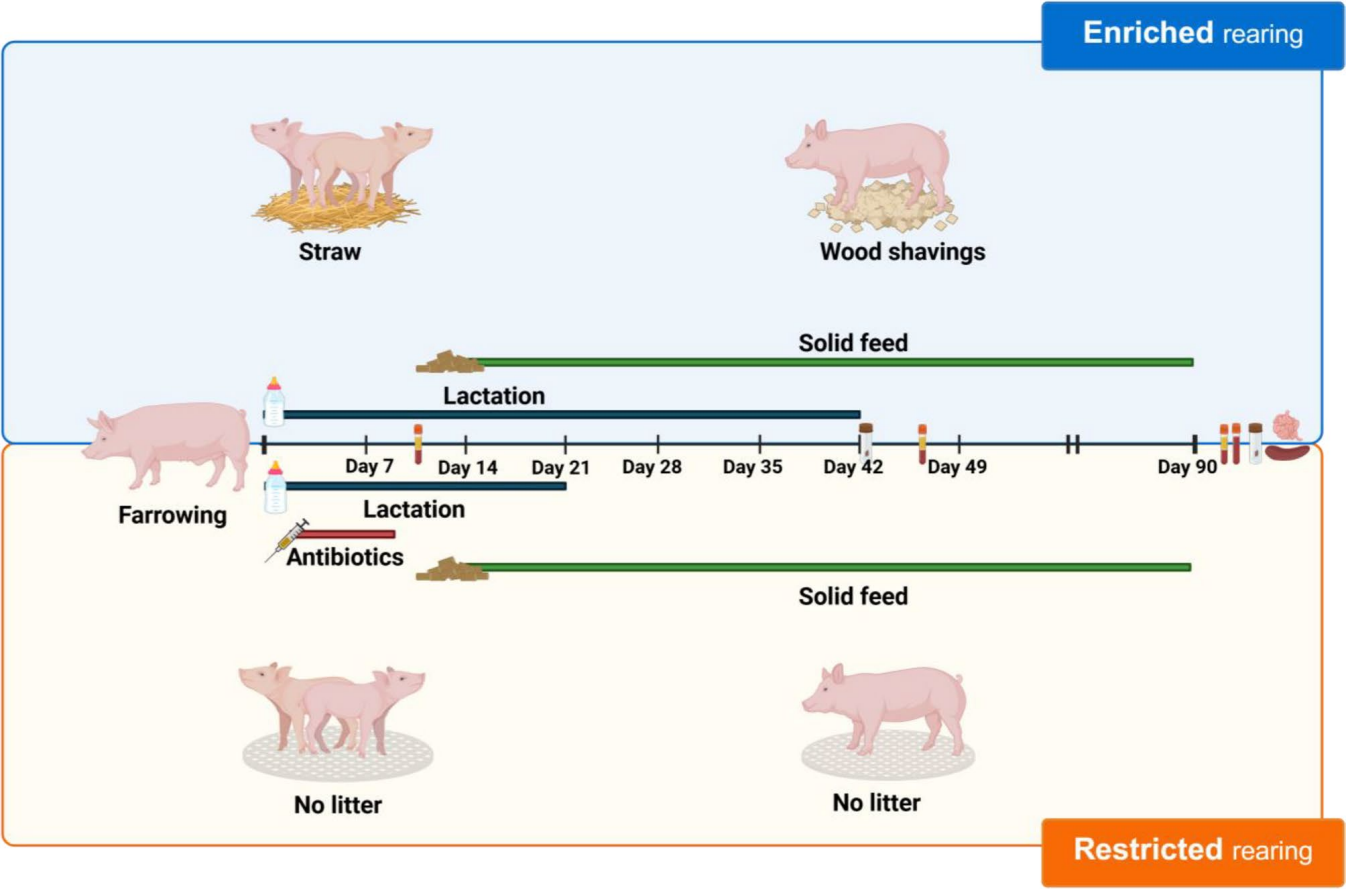
Schematic overview of the differences between enriched and restricted pigs from farrowing to the endpoint of the experiment (day 90). Enriched piglets were weaned at six weeks (42 days) of age, and had litter (straw until weaning, and wood shavings thereafter) for the entirety of the experiment. Restricted piglets were administered antibiotics (days 2-4: trimethoprim-sulfamethoxazole, and days 5-9: amoxicillin) and weaned at three weeks (21 days) of age. Restricted piglets had no litter throughout. Both groups had the same solid feed, provided from week 2 (before weaning) onwards. Serum was taken on days 11, 46, and 90; a rectal swab on day 42; whole blood, intestinal tissue and content, faeces (rectal), and spleen tissue were also collected on day 90

To summarise, weaning, antibiotics and litter material were used to create contrasting, potentially microbiota-restricting and microbiota-enriching conditions, as visualised in Fig. 1.

Finally, 32 animals (16 per group) were chosen for sampling. The final sampling took place on days 91 and 92 (8 pigs from each group per day), hereafter referred to as “day 90” for simplicity. Each group consisted of 8 males and 8 females. Animals were sacrificed after sedation with combined ketamine (20 mg/kg) and Stresnil (azaperone) (2 mg/kg) which was followed by the application of a captive bolt gun (Matador) to the head.

### Sample collection and measurements

Individual bodyweights were measured on days 0, 3, 11, 18, 21, 27, 42, 49, 69 and 90.

Blood was collected from the jugular vein on days 11, 46 and 90. For serum, blood was collected in CAT tubes, and K_2_ EDTA tubes for whole blood. To obtain serum, tubes were centrifuged at 4 °C for 15 min at 3000 × $\:g$. Serum was stored at − 80 °C, and at − 20 °C for short-term storage prior to testing. All samples underwent equal freeze-thaw cycles.

For rectal swabbing (faecal microbiota) on day 42, a sterile specimen collection fluid-1 (SCF-1) solution was prepared to moisten the swabs, in accordance with the Human Microbiome Project’s sampling recommendations [18]. In brief, Tris buffer, ethylenediaminetetraacetic acid, and Tween-20 were combined in ultrapure water. Immediately prior to sampling, sterile swabs (4N6FLOQSwabs™) were moistened with SCF-1 solution and were inserted into the rectum of the piglet before snap-freezing in liquid nitrogen.

On day 90, ileal and colonic content, and rectal faeces were collected and immediately snap-frozen in liquid nitrogen. Content was taken from the ileal midpoint, identified as halfway between the ileo-caecal junction and the distal third of the small intestine; and from the distal portion of the colon. All samples were stored at − 80 °C. For RNA analysis, ileal tissues were rinsed in sterile phosphate-buffered saline. Tissue was taken from the midpoint of the ileum; and for the spleen, from approximately the same portion of the splenic pulp each time. The tissue samples were cut into small pieces and immersed in RNAlater solution (ABPBiosciences, Beltsville, USA). After 24 h at 4 °C, excess RNAlater was poured off, before snap-freezing in liquid nitrogen and storing at − 80 °C. For histomorphology, a section of tissue was cut from the ileum (midpoint) and fixed in 4% formaldehyde solution for 48 h at room temperature. Following fixation, samples were stored in 70% ethanol until processing.

### Haematology

Whole blood analysis was carried out by the university hospital (Universitair Ziekenhuis Leuven, Belgium). Electrical impedance (XN-1000, Sysmex) was used to measure erythrocyte parameters including haemoglobin concentration, mean corpuscular volume (MCV), haematocrit, mean corpuscular haemoglobin (MCH), mean corpuscular haemoglobin concentration (MCHC) the percentage of macro- and microcytic cells, and red cell distribution width – standard deviation (RDW-SD). Leucocyte counts were also performed using the same device, and neutrophil activation indicators (granularity intensity and reactivity intensity) were measured. Total granulocyte counts were calculated as the sum of neutrophils, eosinophils, basophils and immature granulocytes.

### Serum immunoglobulin G measurement

Serum immunoglobulin (Ig) G was measured via nephelometry (IMMAGE 800, Beckman Coulter) by Universitair Ziekenhuis Leuven.

### Ileal histomorphology

Fixed tissues were stained with haematoxylin and eosin, periodic acid-Schiff, and Alcian blue. Staining and scanning of the tissues was performed by GIGA (Liège, Belgium). The digital microphotographs were then analysed using NDP.view2 software (Hamamatsu Photonics K.K., Hamamatsu, Japan). For each animal, the villus height and crypt depth was measured for ten villi and their corresponding crypts of Lieberkühn, with their ratio providing the villus height to crypt depth (VH:CD) ratio. One animal was excluded from the final analysis due to poor histomorphological quality.

### DNA extraction

DNA was extracted from rectal swabs, ileal content and faeces using the QIAamp PowerFecal Pro Plus kit (Qiagen Benelux, Venlo, Netherlands) from 400 mg starting material. Aside from the increased mass of starting material, all other procedures were carried out in accordance with the manufacturer’s instructions. Following extraction, DNA concentration was measured via spectrophotometry (SimpliNano Nanodrop) while DNA structural integrity was assessed via 1% agarose gel electrophoresis. Extracted DNA was stored at − 20 °C.

### DNA sequencing and microbiota analysis

DNA samples underwent Pacific Biosciences (PacBio) V1-V9 16 S rRNA gene sequencing, performed by the VIB Nucleomics Core (Leuven, Belgium). Sequencing data were obtained as FASTQ files. FASTQC was used to perform an initial quality check. Subsequent analysis was performed using QIIME2 with DADA2 for denoising [19], with α-rarefaction revealing sufficient sequencing depth had been attained, and subsequently retaining only sequences ≥ 1000 bp, with no truncation threshold applied. Primers were removed prior to analysis (27 F: AGRGTTYGATYMTGGCTCAG and 1492R: RGYTACCTTGTTACGACTT). A ZymoBIOMICS^®^ community standard was included as a positive control. Taxonomic assignment was performed using the SILVA database (v. 138.99). All QIIME2 steps were executed via a Snakemake workflow on a high-performance computing platform provided by the VSC (Flemish Supercomputer Centre). Following QIIME2, the appropriate outputs were imported into RStudio where subsequent analysis was performed using R.

For α-diversity, the Chao1, Pielou evenness, Shannon and Simpson indices were calculated, with the Wilcoxon test determining significant differences. Bray-Curtis, Sørensen, weighted and unweighted UniFrac distances were plotted for β-diversity. PERMANOVA was used with 9999 permutations to assess statistical significance between groups, with analysis of similarity (ANOSIM) and β-dispersion used as complementary measures. β-dispersion was calculated via the betadisper() R function, with statistical differences judged according to ANOVA. Linear discriminant effect size (LEfSe) analysis was performed in RStudio using the microbiomeMarker package. An LDA score > 3 was chosen as the minimum value for statistically-significant differences.

Six samples were included in a different sequencing run for logistical reasons. These are henceforth excluded from the analysis so as to avoid potential methodological batch effects (*n* = 1 rectal swab, 1 ileum, and 4 faeces [2 per group]).

### Volatile fatty acid measurement

Volatile fatty acid (VFA) measurement was performed according to a modified protocol of Van Craeyveld et al. [20]. In brief, 350 mg ileal, colonic, and faecal matter were weighed, with the exact mass recorded to the nearest 10 mg. Next, 50 µL of a 70.52 mM 2-methylhexanoic acid internal standard was added, before adding 80 µL 6 M hydrochloric acid. Following 20 min in an ice bath with periodic vortexing, 120 µL 25% sodium chloride was added alongside 840 µL *tert*-butyl methyl ether. Following centrifugation at 4 °C for 5 min at 10 000 × $\:g$, the supernatant was added to activated anhydrous sodium sulphate and centrifuged once more as before. Samples were stored at − 20 °C prior to injection into a gas chromatograph (HP 6890 Series). Solutions comprising acetate, propionate, isobutyrate, butyrate, isovalerate, valerate, isocaproate, caproate and enanthate were used to plot standard curves. Sample concentrations were corrected for the exact sample weight and expressed as mM/g accordingly. For analysis, total short-chain fatty acids (SCFAs) were the sum of acetate, proprionate, butyrate, and valerate. Total branched-chain fatty acids (BCFAs) comprised isobutyrate, isovalerate, and isocaproate. Total VFAs comprised all VFAs measured (acetate to enanthate, including BCFAs).

### Faecal water content measurement

Approximately 300 mg frozen faeces was measured, with the exact weight recorded to the nearest milligram. Samples were placed in a freeze-dryer (Christ Alpha 1–4, Osterode am Harz, Germany) for 48 h. Following lyophilisation, the mass of freeze-dried sample was recorded, from which the mass of the pre-weighed, empty Eppendorf tube was subtracted. The water content was the mass of water lost as a percentage of the starting mass.

### Correlation analysis

SparCC, as suited to microbiota data, was used to calculate genus-genus correlations [21]. Taxonomic data at the genus level underwent Dirichlet normalisation and log transformation. SparCC was then performed in Python, using 50 iterations for correlation estimation, and two iterations for the inner convergence loop. Correlations with correlation scores < 0.50 were removed. Statistical significance was determined via 50 permutation iterations to calculate one-sided pseudo *P*-values, with *P* < 0.05 deemed statistically significant.

For genus-metadata correlations, Spearman correlation was performed in RStudio using taxonomic abundance data and the biological metadata. All correlation scores (ρ) had values > 0.50, and FDR correction was applied to *P*-values, with the resultant *q*-values < 0.05 judged statistically significant.

The abovementioned correlation datasets were then combined and plotted as a node-link diagram. Correlations were always performed on the whole cohort (restricted and enriched combined), rather than between-group comparisons. Fisher’s *z*-transformation revealed correlations to have sufficient statistical power (> 0.80).

### RNA extraction

Ileum and spleen tissue samples were thawed and briefly dabbed off to remove excess RNAlater. 20 mg of each tissue was subsequently weighed. RNA extraction was performed using the ReliaPrep™ RNA Tissue Miniprep System kit (Promega, Madison, USA) in accordance with the manufacturer’s instructions. Following extraction, the RNA concentration was measured spectrophotometrically (SimpliNano Nanodrop, Massachusetts, USA). RNA was stored at − 80 °C until use.

### Primer design

A total of 96 genes, including ten housekeeping genes were selected for gene expression analysis (Table S2). Genes were chosen for their immunological roles, with a smaller number of genes involved in folate, vitamin B_12_, and erythrocyte-related functions. Primers were either designed *de novo* via the NCBI Primer-BLAST tool, or obtained from already-published studies. All primers spanned exon-exon junctions and ranged from 70 to 180 nucleotides in length. Primers were produced by IDT DNA (Leuven, Belgium), and their efficiency and specificity was determined by performing real-time PCR (QuantStudio 6 Real-Time PCR Systems, Thermo Fisher Scientific) with a three-fold-diluted cDNA pool from all spleen samples. The Ct values were plotted against the log-transformed cDNA concentration to calculate primer efficiencies. Agarose gel electrophoresis was used to ensure the presence of a single product for each primer.

### Reverse transcription, preamplification and high-throughput qPCR

RNA was processed and quantitative PCR (qPCR) performed following methods previously described by Akram et al. [22]. To summarise, 50 ng RNA was reverse-transcribed using a reverse transcription master mix (Standard BioTools, California, USA). Preamplification was conducted using a Fluidigm PreAmp master mix (Standard BioTools, California, USA) with primers pooled in Tris EDTA buffer which was added to a pooled cDNA sample. Preamplification took place in a 96-well plate at 95 °C for 2 min, 14 cycles at 95 °C for 15 s, followed by 60 °C for 4 min. Exonuclease I (New England Biolabs, Massachusetts, USA) was used to remove any unincorporated primers. Samples were diluted 1:10 in Tris EDTA before storing at − 20 °C.

For qPCR, a sample mix (SSoFast™ EvaGreen^®^ Supermix, low ROX 20X DNA binding dye) and an assay mix (2X assay loading reagent, and 1X DNA suspension buffer) were prepared as described previously [22]. The sample mix and assay mix were then loaded into a 96 × 96 plate and run on a BioMark™ HD instrument. The qPCR procedure comprised denaturation at 95 °C for 60 s and 30 cycles at 96 °C for 5 s, followed by annealing/elongation at 60 °C. The results were visualised and analysed via SBI Real-Time PCR software (Standard BioTools, California, USA).

### Gene expression analysis

Ten housekeeping genes were evaluated for their gene stability via NormFinder [23]. The geometric mean of the expression of the four most stable genes (*HPRT1*,* PGK1*,* GAPDH*, and *RPL4*) was then used to calculate relative gene expression of the non-housekeeping genes via 2^−ΔΔCt^ normalisation. The Wilcoxon test with FDR correction was used as an initial measure to determine significant differences in gene expression between groups. For a small number of samples, some genes had a relative expression of 0 due to the concentration of cDNA being relatively low for pig genes as the procedure had previously been optimised for chicken samples [22]. For these 0 values, K-nearest neighbours imputation was performed (*k* = 5) using the R package impute. This imputation however did not affect the number of genes with *P* < 0.05.

Next, gene set enrichment analysis (GSEA) was performed via the GSEA application (v. 4.3.3) to find gene sets with different expression patterns between the two groups. An initial nominal *P*-value < 25% was considered significant as is standard for GSEA. FDR correction was then performed to provide a *q*-value, and genes within these gene sets contributing significantly to the expression patterns were identified as core enrichment genes.

### Statistics

Statistical tests were performed as described hitherto, appropriate for each individual analysis, and the normality of data distribution as determined via the Shapiro-Wilk test. For all tests, *P* < 0.05 was deemed statistically significant, while *P* < 0.10 was interpreted as a trend towards significance.

Further statistical analysis was performed to explore the potential effects of the litter of origin on the observed outcomes, as opposed to the experimental group itself. Linear mixed-effects modelling, and generalised linear mixed modelling was performed, the former for data whose residuals were normally distributed, and the latter for non-normally distributed datasets. Likelihood ratio testing was then performed to determine whether the inclusion of litter of origin led to a significant difference in the modelling alongside group, as opposed to group as the sole factor.

## Results

### Bodyweight

**Fig. 2:**
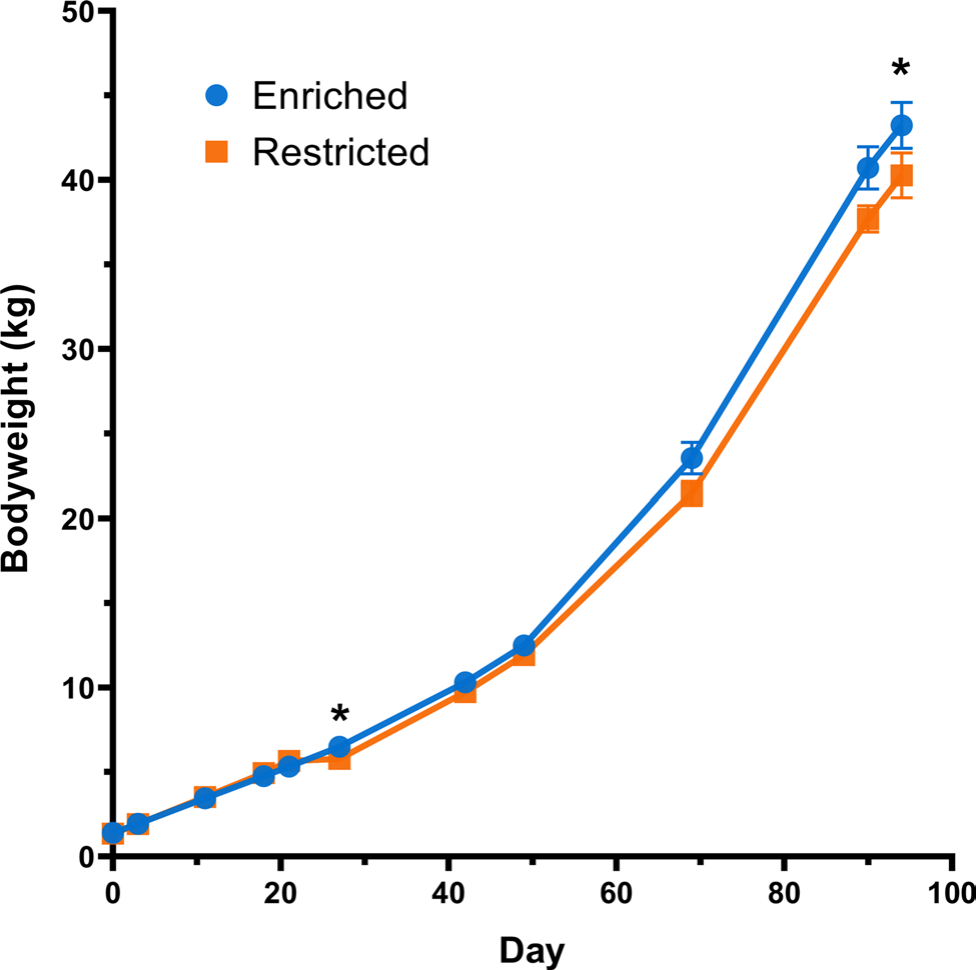
Mean bodyweight by group from farrowing (day 0) to the endpoint of the experiment (day 90). Mean bodyweight is plotted for each group, with the standard error of the mean shown. The asterisks denote the two timepoints at which the bodyweight was significantly different by group according to the Wilcoxon test. *N* = 32 per group

Significant differences were observed in the bodyweight on days 27 and 90 with enriched pigs exhibiting significantly higher mean bodyweights than their restricted counterparts (*P* = 0.041 and 0.044 respectively) (Fig. 2, Table S3). Day 69 also indicated a trend in the same direction (*P* = 0.080).

Of note, while weaning at day 21 led to bodyweight increasing on average by just 0.12 kg (2.1%) among the restricted group, weaning at day 42 appeared not to perturb bodyweight gain with the enriched group gaining on average 2.18 kg (21.2%) the following week.

### Microbiota analysis

#### β-diversity

**Fig. 3:**
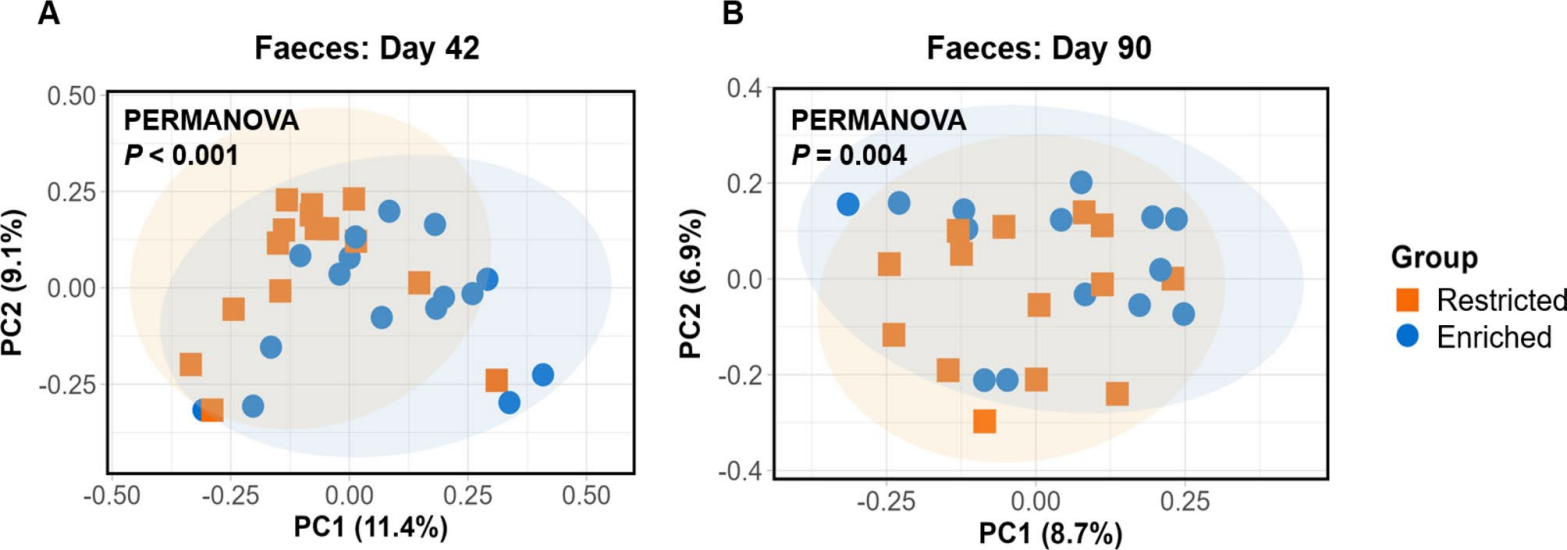
Principal coordinate analysis (PCoA) of β-diversity of the faecal microbiota on day 42 (**A**) and day 90 (**B**). Both graphs display β-diversity calculated according to the Bray-Curtis index, with PERMANOVA indicating significant differences between groups at both timepoints (*P* < 0.001, R^2^ = 0.059, and *P* = 0.004, R^2^ = 0.051 for days 42 and 90 respectively)

At 42 days of age, β-diversity differed significantly among the two groups for the Bray-Curtis (Fig. 3) and Sørensen (Figure S1) distances according to PERMANOVA. On the other hand, neither of the UniFrac distances showed significant differences (Figure S1).

On day 90, β-diversity did not differ by group for the ileal microbiota. Nevertheless, for the faeces, there were significant differences for the Bray-Curtis (Fig. 3) and Sørensen distances (Figure S2), with a trend also observed with unweighted UniFrac distance (Figure S2). All significant PERMANOVA results were verified as significant by ANOSIM. For significantly-different distance metrics, no significant intragroup differences were found in terms of β-dispersion (*P* > 0.05).

#### α-diversity

No significant differences in α-diversity were found between the two groups’ faecal microbiota at day 42. Likewise, there were no significant differences in the ileum at day 90, although the restricted group did exhibit a trend towards higher diversity for the Chao1 index (*P* = 0.063). In day 90 faecal samples, the enriched group exhibited significantly higher α-diversity for both the Pielou evenness and Simpson indices (*P* = 0.003 and 0.019 respectively) (Figure S3). Of note, α-diversity values for the ileum were consistently lower than those of the day 42 and day 90 faeces, irrespective of group.

#### Taxonomic composition

Across all 90 samples, a total of 11 269 ASVs were found, which amounted to 21 phyla and 385 genera. All were bacteria, with no archaea detected. Of all phyla, Firmicutes predominated in the faeces on day 42, with a mean abundance of 48.6%, followed by Bacteroidetes at 23.9% and Spirochaetes at 7.6%. By day 90, Firmicutes had increased in abundance in the faeces to 68.4%, again followed by Bacteroidetes (28.5%), with reduced Spirochaetes (0.9%). The ileum, representing a less-diverse microbial environment, was heavily dominated by Firmicutes (97.1%), with Actinobacteria (2.2%) and Proteobacteria (0.5%) compromising the next most abundant phyla.

**Fig. 4:**
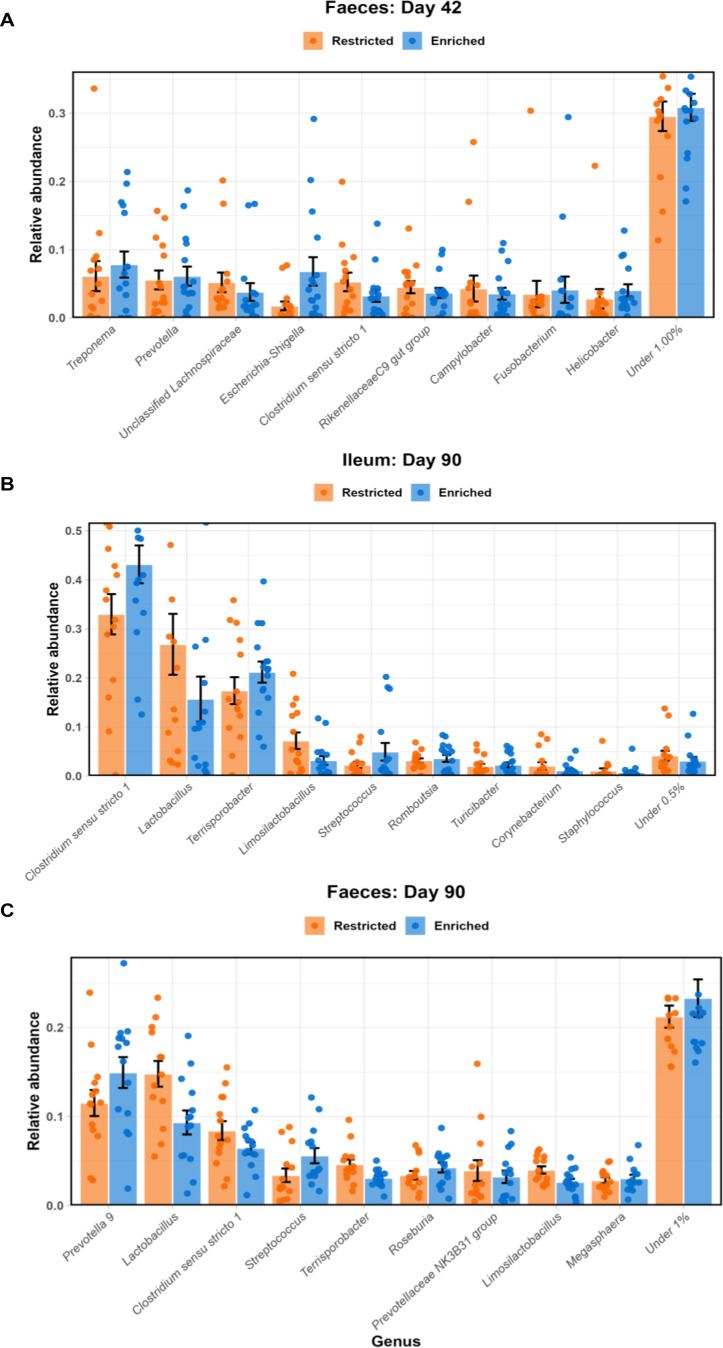
Mean taxonomic abundance per group at the genus level for faeces day 42, ileum day 90 and faeces day 90. All three graphs depict the 10 most abundant genera according to the mean relative abundance. Error bars depict the standard error of the mean per group. Graph A shows the faecal microbiota (*n* = 15 restricted, 16 enriched) on day 42, with taxa with a mean abundance of < 1.0% grouped together. Graph B shows the ileal microbiota (*n* = 15 restricted, 16 enriched) on day 90, with taxa with a mean abundance of < 0.5% grouped together, while graph C shows the faecal microbiota (*n* = 14 per group) on day 90, with taxa with a mean abundance of < 1.0% grouped together

At the genus level, no single genus had a mean abundance > 7% on day 42 in the faeces (Fig. 4, S4), with *Treponema* exhibiting an abundance of 7.0%. This was closely followed by *Prevotella*, unclassified Lachnospiraceae and *Escherichia-Shigella* at 5.9%, 4.5% and 4.4% respectively. By day 90, *Prevotella 9* and *Lactobacillus* had become the two most abundant genera in the faeces with mean abundances of 13.2% and 12.1% respectively, with *Clostridium sensu stricto 1* and *Streptococcus* following with abundances of 7.4% and 4.5%. The ileum on the other hand, like with its phyla, was dominated more heavily by a smaller number of taxa. *Clostridium sensu stricto 1* comprised on average 38.2% of ileal genera, with *Lactobacillus*,* Terrisporobacter* and *Limosilactobacillus* exhibiting abundances of 21.1%, 19.4% and 5.1% respectively.

#### LEfSe analysis between groups

**Fig. 5:**
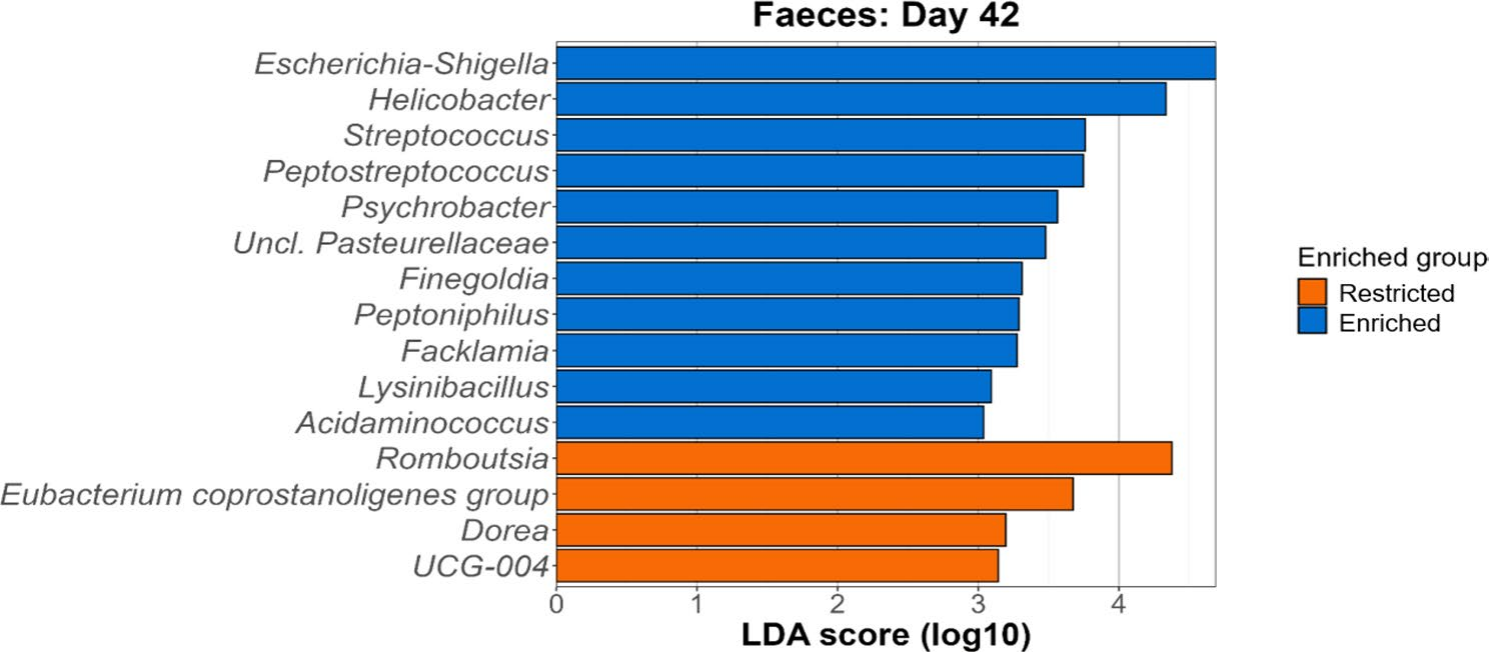
Genera enriched among the restricted and enriched groups on day 42 (faeces) as identified by LEfSe analysis. For each genus deemed significantly different between the two groups, the LDA score is visualised on the $\:x$-axis, with a threshold of > 3 chosen for a significant difference

Comparing the restricted and enriched groups, 15 genera were deemed significantly different in abundance in day-42 faeces, according to LEfSe analysis (Fig. 5). The microbiota of restricted piglets was found for instance to be characterised by *Romboutsia*,* Dorea* and *UCG-004* (family Erysipelatoclostridiaceae). On the contrary, enriched piglets exhibited higher levels of *Escherichia-Shigella*,* Helicobacter*,* Streptococcus* and *Peptostreptococcus*.

**Fig. 6:**
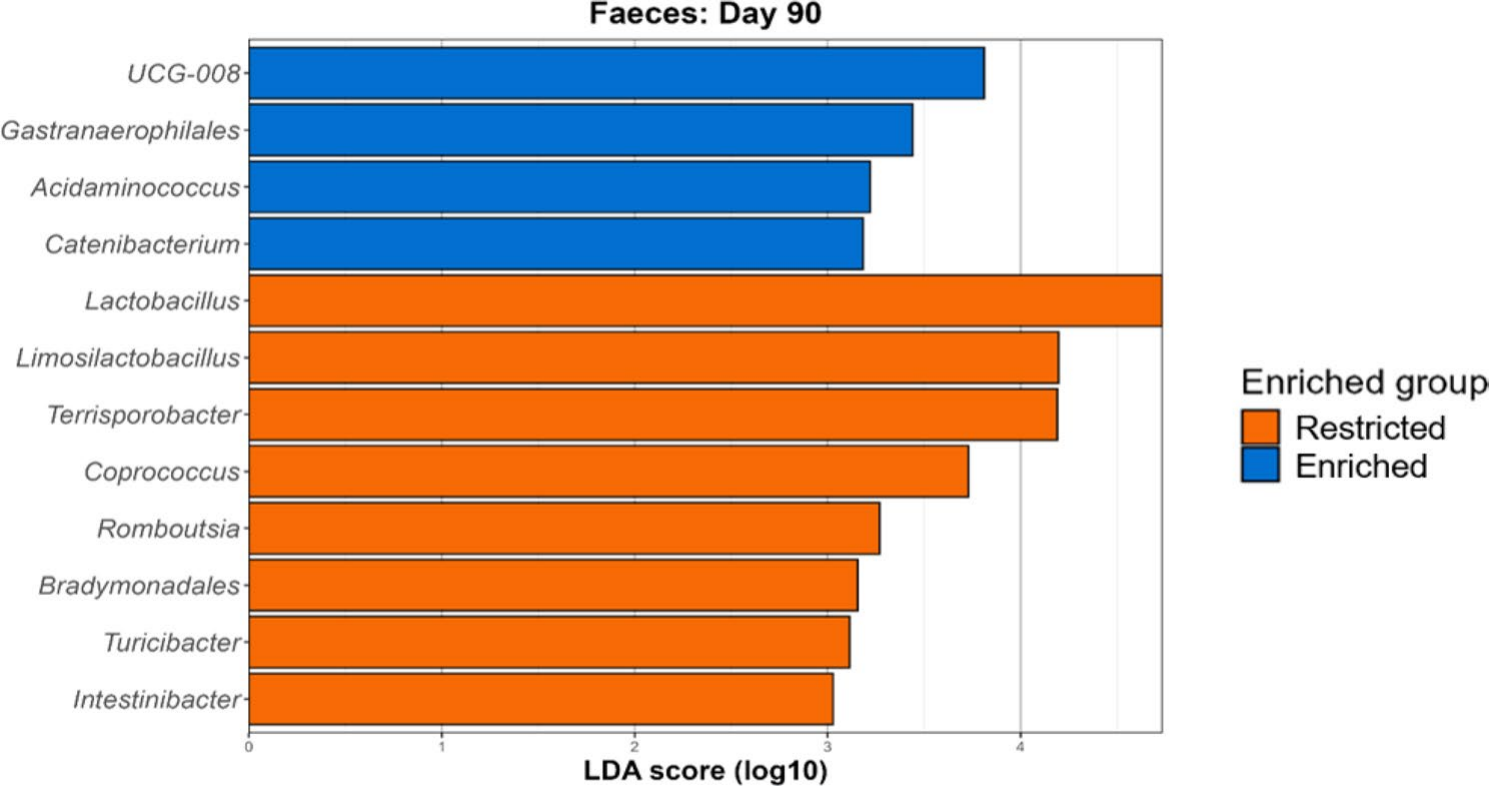
Genera enriched among the restricted and enriched groups on day 90 (faeces) as identified by LEfSe analysis. For each genus deemed significantly different between the two groups, the LDA score is visualised on the $\:x$-axis, with a threshold of > 3 chosen for a significant difference

On day 90, four genera were found to be significantly different in the ileal microbiota. *Limosilactobacillus*, unclassified Pasteurellaceae and *Escherichia-Shigella* were typical of restricted pigs, while *Enterococcus* served as a marker for the enriched pigs (Figure S5). In the faeces however, 12 genera were significantly different between the two cohorts (Fig. 6). The lactic acid bacteria *Lactobacillus* and *Limosilactobacillus* were elevated in the faeces of restricted pigs, alongside *Terrisporobacter*,* Coprococcus*,* Romboutsia*,* Turicibacter*,* Bradymondales* and *Intestinibacter*. *UCG-008* (family Butyricicoccaceae), *Gastranaerophilales*,* Acidaminococcus* and *Catenibacterium* were on the other hand characteristic of the enriched pigs’ microbiota.

### Volatile fatty acids

On day 90, no significant differences were observed in ileal VFAs (Table 1 & Figures S6-8). In the faeces, total BCFAs were higher in the restricted group in comparison to the enriched pigs. Moreover, butyrate was also shown to be significantly elevated in the faeces of the restricted pigs on day 90. Likewise, colonic isobutyrate, isovalerate, and total BCFAs were significantly higher in restricted piglets on day 90. However, it should be noted that nominal *P*-values did not retain significance after FDR correction.

**Table 1:** Volatile fatty acid compositions between restricted and enriched pigs across the ileum, colon and faeces on day 90

	Ileum					Colon					Faeces				
	Restricted		Enriched		*P*-Value	Restricted		Enriched		*P*-Value	Restricted		Enriched		*P*-Value
	Mean (mM/g)	SEM	Mean (mM/g)	SEM		Mean (mM/g)	SEM	Mean (mM/g)	SEM		Mean (mM/g)	SEM	Mean (mM/g)	SEM	
Acetate	50.53	4.81	54.16	7.01	0.953	200.19	8.28	197.91	6.05	0.770	170.64	8.50	147.52	8.14	0.074
Propionate	7.73	0.58	6.73	1.00	0.752	86.13	3.72	90.65	4.53	0.232	65.35	3.89	61.67	4.68	0.437
Isobutyrate	0.62	0.10	0.41	0.11	0.185	3.50	0.23	3.12	0.29	**0.045**	5.81	0.48	4.98	0.61	0.056
Butyrate	1.17	0.06	1.31	0.17	0.830	55.87	2.70	56.66	3.54	0.572	45.79	2.73	38.67	2.61	**0.048**
Isovalerate	0.96	0.17	0.83	0.16	0.526	3.78	0.31	3.28	0.38	**0.037**	6.90	0.61	6.08	0.81	0.096
Valerate	0.08	0.08	0.00	0.00	**-**	8.32	0.59	10.17	1.18	0.318	6.68	0.50	6.60	0.70	0.341
Isocaproate	0.00	0.00	0.00	0.00	**-**	0.27	0.08	0.26	0.08	0.812	0.36	0.08	0.41	0.08	0.720
Caproate	0.07	0.07	0.00	0.00	**-**	1.65	0.15	1.70	0.11	0.711	1.76	0.15	1.65	0.11	0.508
Enanthate	0.00	0.00	0.00	0.00	**-**	4.37	1.39	5.20	1.39	0.918	0.77	0.12	0.89	0.09	0.725
Total SCFAs	59.51	4.93	62.21	7.61	0.985	350.51	13.17	355.39	12.27	0.401	288.46	14.80	254.47	15.24	0.108
Total BCFAs	1.57	0.27	1.24	0.26	0.267	7.55	0.52	6.65	0.67	**0.030**	13.07	1.09	11.47	1.43	**0.048**
Total VFAs	61.16	5.05	63.45	7.77	0.985	364.08	12.66	368.94	12.21	0.572	304.05	14.88	268.47	15.69	0.122

The mean concentrations are indicated ± the standard deviation of the mean. Significant *P*-values (nominal values) are indicated in bold, as calculated with the Wilcoxon test. For the ileum, *n* = 15 restricted, 16 enriched, colon *n* = 15 restricted, 16 enriched, and faeces *n* = 14 per group samples were used for final analysis. (Total SCFAs: acetate, propionate, butyrate, and valerate. Total BCFAs: isobutyrate, isovalerate, and isocaproate. Total VFAs: acetate, propionate, isobutyrate, isovalerate, valerate, isocaproate, caproate, and enanthate)

Data are presented as violin plots in Figures S4-6

### Faecal water content

No significant differences were found in terms of faecal water content, although there was a trend for the restricted group having reduced water content (*P* = 0.096). Water content amounted to an average of 76.77% ± 0.65 SEM and 78.34% ± 0.60 for the restricted and enriched groups respectively.

### Blood analysis

#### Serum IgG over time

**Fig. 7:**
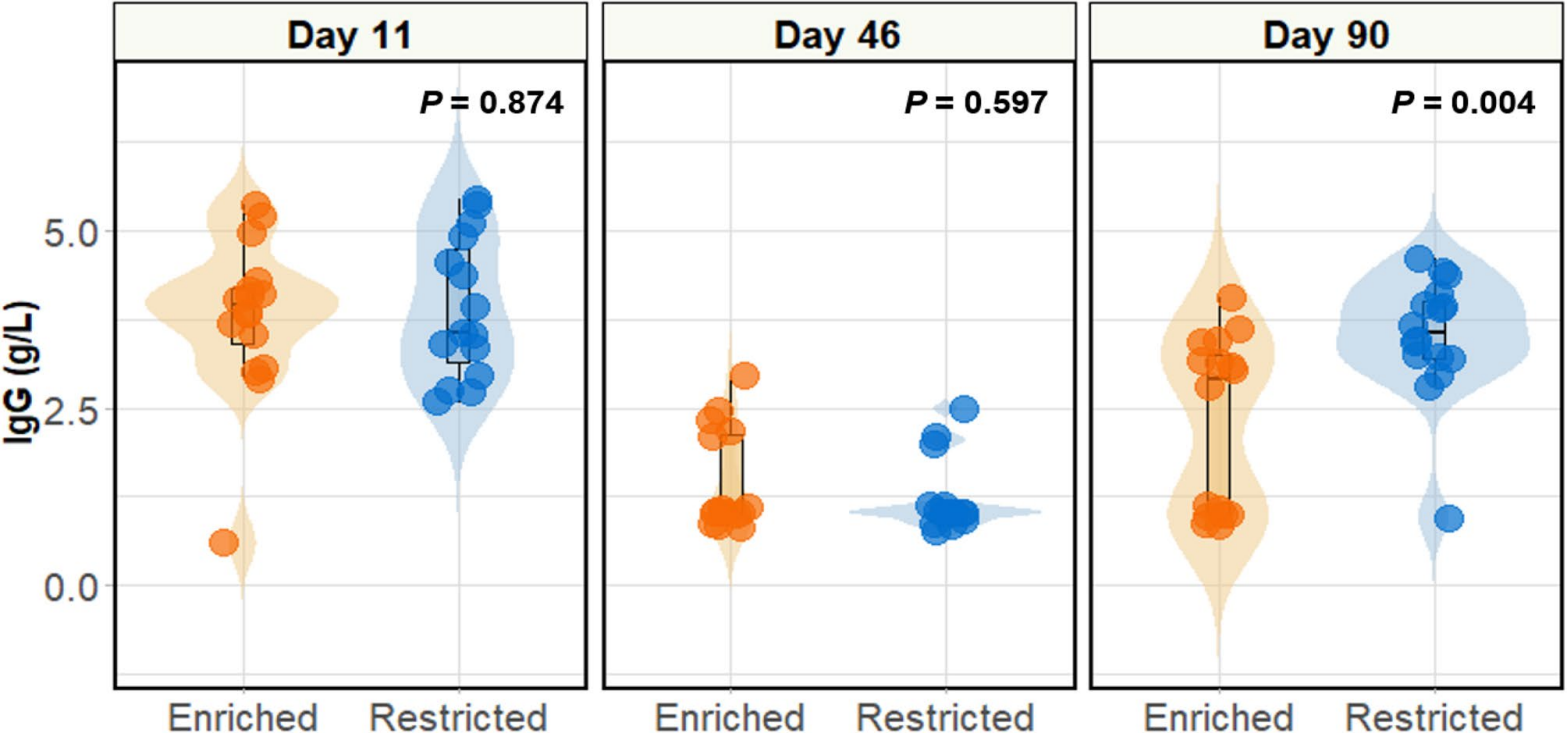
Serum immunoglobulin G levels on days 11, 46 and 90. Violin plots depict serum IgG by group for the three timepoints. The Wilcoxon test identified statistically significant differences on day 90 (*P* = 0.004). For day 11, *n* = 15 restricted, 16 enriched, day 46 *n* = 16 per group, and day 90 *n* = 16 per group

No significant differences were found in serum IgG levels between the two groups on days 11 or 46 (Fig. 7). Nonetheless, restricted pigs showed significantly higher IgG levels on day 90 compared to their enriched counterparts (*P* = 0.004).

#### Erythrocyte parameters

On day 90, a number of differences were observed between the two groups when comparing erythrocyte-related parameters (Table 2). Firstly, MCV was significantly elevated among enriched pigs, as was the percentage of erythrocytes classified as macrocytic, while the restricted group exhibited significantly more microcytic cells. RDW-SD was also significantly higher for the enriched group, indicating that these pigs exhibited greater variation in erythrocyte size.

**Table 2:** Erythrocyte parameters on day 90

Parameter	Unit	Restricted		Enriched		*P*-Value
		Mean	SEM	Mean	SEM	
Erythrocytes	10^6^/µL	6.64	0.14	6.51	0.20	0.590
Haemoglobin	g/dL	10.73	0.20	11.38	0.59	0.610
MCV	fL	64.02	0.59	67.12	0.77	**0.004**
Haematocrit	NA	0.43	0.01	0.44	0.02	0.571
MCH	pg	16.29	0.18	16.71	0.18	0.104
MCHC	g/dL	25.30	0.22	24.93	0.34	0.597
Macrocytic erythrocytes	%	0.75	0.06	1.08	0.09	**0.017**
Microcytic erythrocytes	%	45.88	2.09	36.28	1.79	**0.004**
RDW-SD	NA	41.06	0.55	43.60	1.24	**0.042**

Erythrocyte count, haemoglobin concentration, mean corpuscular volume (MCV), haematocrit, mean corpuscular haemoglobin (MCH), mean corpuscular haemoglobin concentration (MCHC), the percentage of macrocytic erythrocytes, the percentage of microcytic erythrocytes, and red cell distribution width – standard deviation (RDW-SD). Values are provided as means per group ± standard error of the mean. *P*-values were calculated according to the Wilcoxon test. *N* = 16 per group

#### Leucocyte counts

When comparing leucocyte counts on day 90, no significant differences were found between groups for total leucocytes, granulocytes or neutrophil counts (Table 3). On the other hand, restricted pigs displayed a trend towards higher lymphocyte counts (*P* = 0.097), while enriched pigs exhibited a trend towards elevated monocytes (*P* = 0.092). Furthermore, as percentages of the total leucocytes, granulocytes and neutrophils were higher in enriched pigs, while lymphocytes were elevated in the restricted group. The neutrophil to lymphocyte (N:L) ratio was also significantly elevated in the enriched group compared to restricted pigs (*P* = 0.003).

**Table 3:** Leucocyte parameters at day 90

Parameter	Unit	Restricted		Enriched		*P*-Value
		Mean	SEM	Mean	SEM	
Total leucocytes	10^3^/µL	18.14	1.42	17.38	1.33	0.696
Total granulocytes	10^3^/µL	8.15	0.90	9.46	0.89	0.268
	%	44.66	3.52	52.03	1.54	**0.020**
Neutrophils	10^3^/µL	7.79	0.81	9.08	0.88	0.213
	%	43.82	2.27	51.25	1.53	**0.031**
Lymphocytes	10^3^/µL	9.17	1.03	6.90	0.50	0.097
	%	50.13	2.48	40.54	1.86	**0.003**
Monocytes	10^3^/µL	0.68	0.11	1.40	0.40	0.092
	%	4.33	0.75	5.78	0.73	0.149
N:L ratio	NA	0.91	0.08	1.34	0.11	**0.003**

Total leucocyte count, total granulocytes (neutrophils + basophils + eosinophils + immature granulocytes), neutrophils, lymphocytes, monocytes, and the neutrophil to lymphocyte (N:L) ratio. Granulocytes, neutrophils, lymphocytes, monocytes are shown as total counts and as percentages of total leucocytes. Values are provided as means per group ± standard error of the mean. *P*-values were calculated according to the Wilcoxon test. *N* = 16 per group

#### Neutrophil activation measures

Haematology results also suggested that neutrophils were more activated in the restricted group compared to the enriched group. This was indicated through both the neutrophil granularity intensity (restricted: 146.89 SI ± 1.57 SEM, enriched: 143.16 SI ± 1.12, *P* = 0.044), and neutrophil reactivity intensity (restricted: 38.35 FI ± 0.47, enriched 36.61 FI ± 0.56, *P* = 0.027).

### Ileal histomorphology

The enriched group exhibited a significantly greater ileal VH:CD ratio than the restricted group on day 90 (*P* = 0.013) (Table 4). On the other hand, no significant differences were found for the villus heights and crypt depths alone, although there was a trend towards greater villus height among enriched pigs.

**Table 4:** Histomorphological parameters of the ileum on day 90

	Restricted		Enriched		*P*-Value
	Mean	SEM	Mean	SEM	
Villus height (µm)	433.23	12.20	470.71	16.99	0.091
Crypt depth (µm)	303.61	8.90	289.55	4.31	0.188
VH:CD	1.49	0.04	1.69	0.06	**0.013**

Values are provided as means per group ± standard error of the mean. *P*-values were calculated according to ANOVA. *N* = 16 restricted, 15 enriched

### Microbiota and metadata correlations

**Fig. 8:**
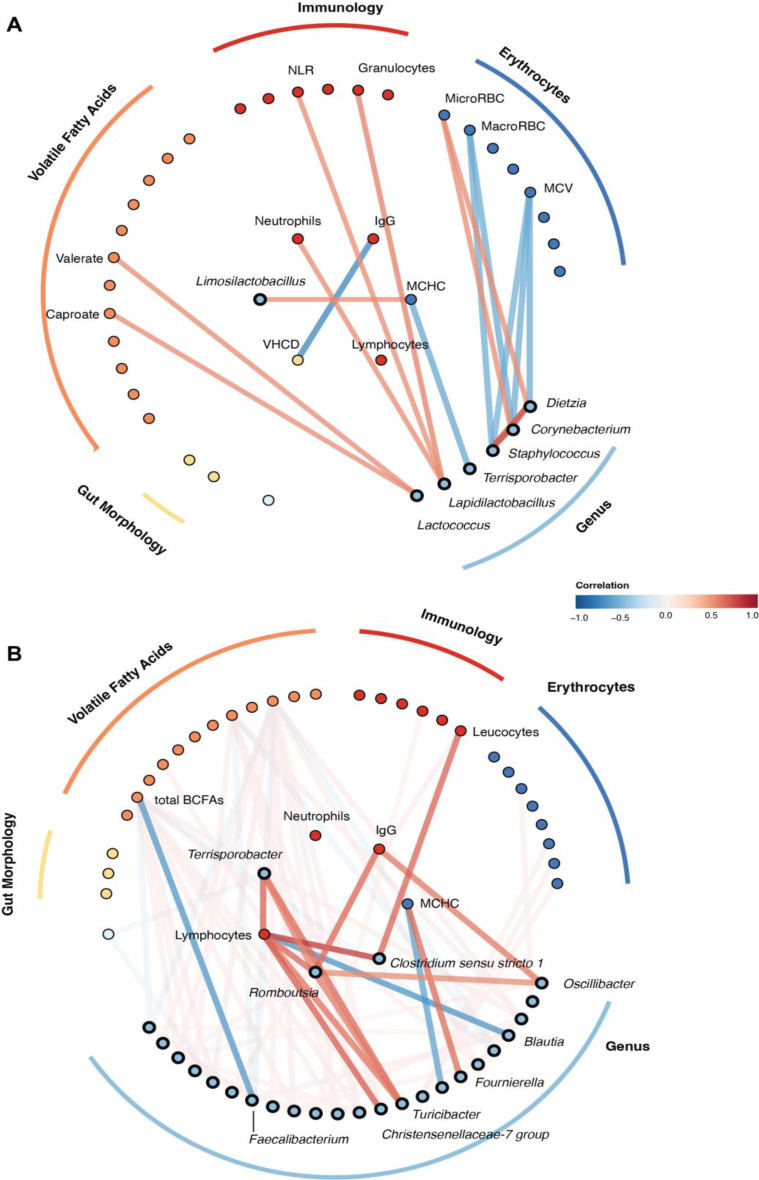
Node-link diagram depicting correlations between ileal (**A**) and faecal (**B**) microbiota components on day 90, and biological metadata. Positive correlations between nodes are linked by a red line, while negative correlations are indicated in blue. Genus-genus correlations visualised were found to be significant through SparCC, with genus-metadata correlations being via Spearman correlation. Nodes of interest are labelled, with those of particular focus in the analysis placed in the centre. NLR represents the neutrophil to lymphocyte (N:L) ratio, MCHC mean corpuscular haemoglobin concentration, microRBC microcytes, macroRBC macrocytes, MCV mean corpuscular volume, IgG immunoglobulin G, and total BCFAs total branched-chain fatty acids. *N* = 31 (A), *n* = 14 per group (B)

Due to limited systemic biological metadata at day 42, the day 90 results were utilised for microbiota-metadata correlations (Fig. 8). The ileal microbiota was found to be significantly correlated with a number of haematological components. MCHC was negatively correlated with *Terrisporobacter* (*q* = 0.014, ρ = −0.576) and positively with *Limosilactobacillus* (*q* = 0.043, ρ = 0.520). Additionally, *Staphylococcus* was positively correlated with microcytes (*q* = 0.042, ρ = 0.521), and negatively with macrocytes and MCV (*q* = 0.031 ρ = −0.548, and *q* = 0.027 ρ = −0.547 respectively). *Corynebacterium*, being positively correlated with *Streptococcus* (correlation = 0.810), was also negatively correlated with macrocytes and MCV (*q* = 0.032 and 0.037, ρ = −0.547 and −0.532 respectively). VH:CD was also strongly negatively correlated with serum IgG (*q* < 0.001, ρ = −0.742). Furthermore, *Lapidilactobacillus* was positively correlated with granulocyte counts, neutrophil counts and the N:L ratio (*q* = 0.019, 0.048, and 0.049, ρ = 0.572, 0.523, and 0.523 respectively). *Lactococcus* was also positively correlated with the ileal VFAs valerate and caproate (*q* = 0.039, ρ = 0.525 for both).

Moving on to the faeces at day 90, blood MCHC was positively correlated with *Fournierella*, while being negatively correlated with *Candidatus Soleaferrea* (*q* = 0.022 and 0.033, ρ = 0.631 and −0.607 respectively). The genera *Romboutsia*,* Terrisporobacter*, and *Turicibacter*, also positively correlated with each other (all correlations > 0.560), were positively correlated with lymphocyte counts (*q* = 0.018, 0.015, and 0.037, ρ = 0.653, 0.659, and 0.610 respectively). *Clostridium sensu stricto 1* was also strongly positively correlated with lymphocytes (*q* < 0.001, ρ = 0.805). Conversely, *Blautia* and *Faecalibacterium* were negatively correlated with lymphocytes (*q* = 0.011 and 0.041, ρ = −0.677 and −0.603 respectively). *Romboutsia* was also positively correlated with IgG (*q* = 0.015, ρ = 0.650), as was *Oscillibacter* (*q* = 0.044, ρ = 0.590). *Faecalibacterium* was additionally negatively correlated with BCFAs (*q* = 0.027, ρ = −0.661).

### Gene expression

#### Principal component analysis

**Fig. 9:**
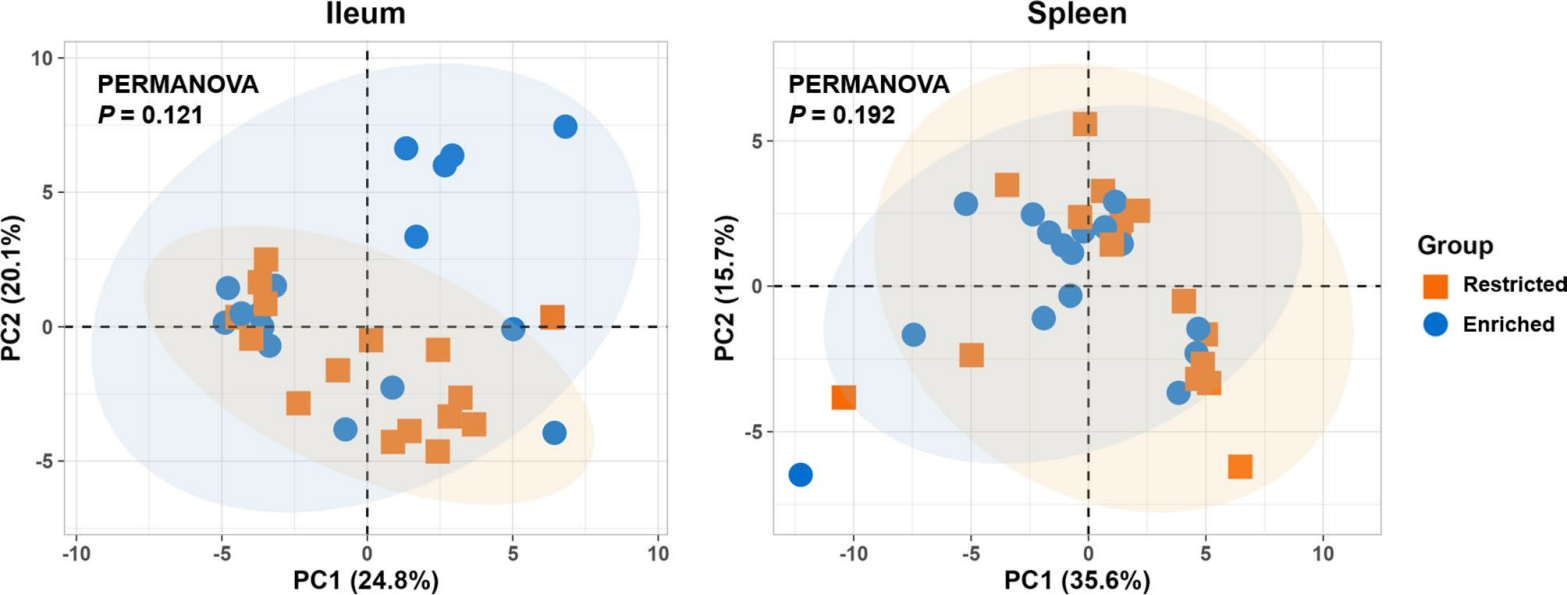
Principal component analysis (PCA) of gene expression profiles in the ileum and spleen on day 90. PCA illustrates the variance between the two groups for both the ileum and spleen. *P*-values were calculated according to PERMANOVA, applying cosine distance. *N* = 16 per group for both tissues

For genes in both the ileum and spleen, PCA did not show distinct clustering patterns by group, as confirmed by PERMANOVA (*P* = 0.121 and 0.192, R^2^ = 0.059 and 0.048 respectively) (Fig. 9). For ileal genes, a number of enriched individuals were visibly more spread out in the PCA plot in comparison with the more tightly-grouped restricted pigs, although there were no significant differences in dispersion between samples.

### Differential gene expression in the ileum

No significant differences were found to satisfy FDR correction. However, according to non-FDR *P*-values, *NLRP3* (NLR family pyrin domain containing 3) and *HMOX1* (haem oxygenase 1) were upregulated in restricted pigs while *TNFA* (tumour necrosis factor alpha) and *NFKBIA* (nuclear factor of kappa light polypeptide gene enhancer in B-cells inhibitor alpha) were upregulated in enriched pigs (*P* < 0.05). Additionally, the genes *GATA3* (GATA-binding protein 3), *CCL2* (C-C motif chemokine ligand 2), and *CCL5* (C-C motif chemokine ligand 5) exhibited a trend towards higher expression in restricted pigs, while *TLR4* (Toll-like receptor 4) tended to be upregulated in the enriched group (*P* < 0.10).

While GSEA found 6 gene sets upregulated for both groups, none of these gene sets were significant in accordance with the FDR *q*-value (data not shown).

### Differential gene expression in the spleen

As with the ileum, differences were not FDR-significant. Nevertheless, the genes *CD83* (cluster of differentiation 83), *FTH1* (ferritin heavy chain 1), *NOD2* (nucleotide-binding oligomerisation domain-containing 2), and *CLEC7A* (C-type lectin domain-containing 7 A) were upregulated in the enriched group according to nominal *P*-values, with *CD320* (cluster of differentiation 320) expression higher in restricted pigs (*P* < 0.05). Furthermore, *IGHE* (immunoglobulin heavy constant epsilon), *TLR5* (Toll-like receptor 5), *IL11RA* (interleukin 11 receptor subunit alpha), *IFNA1* (interferon alpha 1), and *IFNW1* (interferon omega 1) exhibited trends in upregulation in enriched pigs, with *MAPK14* (mitogen-activated protein kinase 14) and *IL6* (interleukin 6) showing higher expression in the restricted group (*P* < 0.10).

GSEA revealed that the pro-inflammatory and chemokine gene sets were significantly upregulated in the restricted group (*q* = 0.104 and 0.232 respectively) (Table 5). Of the pro-inflammatory genes, eight were indicated as core enrichment genes: *CCL2*,* IL15* (interleukin 15), *MAPK14*,* IL6*,* MYD88* (myeloid differentiation primary response 88), *CXCL10* (C-X-C motif chemokine ligand 10), *IFNG* (interferon gamma) and *NFKB1* (nuclear factor kappa B subunit 1), as ranked in descending order of their metric scores. For the chemokine genes, three genes were flagged as core enrichment genes: *CCL2*,* CXCL13* (C-X-C motif chemokine ligand 13) and *CXCL10*.

**Table 5:** Gene set enrichment analysis (GSEA) gene sets significantly upregulated in the restricted group

Gene set			Associated genes			
Name	Nominal *P*	FDR q	Gene	Rank metric	Running ES	Core enrichment
Pro-inflammatory	0.041	0.104	*CCL2*	0.407	0.158	**Yes**
			*IL15*	0.344	0.269	**Yes**
			*MAPK14*	0.337	0.400	**Yes**
			*IL6*	0.283	0.510	**Yes**
			*MYD88*	0.214	0.570	**Yes**
			*CXCL10*	0.189	0.597	**Yes**
			*IFNG*	0.146	0.608	**Yes**
			*NFKB1*	0.116	0.630	**Yes**
			*IL18*	0.011	0.494	No
			*CCL5*	−0.040	0.463	No
			*TNFA*	−0.126	0.373	No
			*IFNA1*	−0.137	0.356	No
			*IL33*	−0.220	0.140	No
Chemokines	0.105	0.232	*CCL2*	0.407	0.420	**Yes**
			*CXCL13*	0.197	0.486	**Yes**
			*CXCL10*	0.189	0.681	**Yes**
			*CCL5*	−0.040	0.448	No
			*CCR7*	−0.136	0.412	No

GSEA results showing the pre-defined gene sets of pro-inflammatory and chemokine-encoding genes deemed significant according to the standard GSEA threshold (*q* < 25%). Rank metric is shown for each gene, indicating its correlation with the treatment group. The running enrichment score (ES) is also shown, and indicates the cumulate enrichment score whereby genes before the peak ES have the greatest contribution to the gene set

### Effect of litter of origin

Given that the experimental design required piglets to remain with their sow under specific pen conditions (litter material), it was not possible for piglets from the same litter to be allocated to different groups. As such, experimental groups and litter of origin were confounded and their effects challenging to distinguish. Nevertheless, additional analysis suggested some potential further effect of litter of origin on the microbiota and physiology into later life. In terms of the microbiota, a nested PERMANOVA of Bray-Curtis β-diversity values revealed increased values for R^2^ when both the litter of origin and experimental group were taken into account (R^2^ = 0.149 and 0.142, *P* < 0.001) for the faecal microbiota on days 42 and 90 respectively.

Additionally, generalised linear mixed models showed litter of origin to have a significant impact on serum IgG on days 46 and 90 (*P* = 0.013 and < 0.001 respectively). Models for other immunological and haematological parameters however showed no significant difference when taking litter into account.

Linear mixed-effects modelling also showed a significant impact of litter of origin when analysing ileal histomorphology, for villus height and VH:CD (*P* = 0.002 and 0.015 respectively).

Lastly, a nested PERMANOVA performed on PCA of gene expression revealed significant differences between the two cohorts when litter was accounted for alongside experimental group, for both the ileum and the spleen (R^2^ = 0.236 and 0.210, *P* = 0.005 and 0.011 respectively). Of all aforementioned genes, including litter of origin in generalised linear mixed modelling proved significant for ileal *HMOX1*,* TNFA*, and *TLR4*, and splenic *CD83*,* FTH1*,* CD320*,* IL15*, and *CCL2*.

## Discussion

As demonstrated by the current findings, early-life rearing conditions can lead to long-term effects on the porcine microbiota, physiology and immunology until day 90. These findings are in accordance with previous work analysing the effects of weaning age [24, 25], antibiotic use [26], and the provision of bedding material [13, 14]. Furthermore, these long-term effects, while in accordance with human infant studies [27], contradict previous studies which found relatively short-lived effects on the microbiota of pigs from environmental changes [25]. Nonetheless, the current study does corroborate previous work on the immunological impacts of early-life changes, such as Schokker et al.’s work on antibiotic-induced disruption of the microbiota [28, 29].

### Weaning and bodyweight

In nature, wild boar wean their young between 12 and 16 weeks of age [30], with domestic sows also doing so naturally by around 12 weeks [31, 32]. Despite being considerably below the natural weaning age, the enriched group’s weaning at 6 weeks did not lead to any decrease, or stagnation in bodyweight, whereas the restricted piglets’ weaning led to poor bodyweight gain the following week. While data on such a prolonged weaning age remain scarce, these findings are in accordance with the general trend found in other weaning studies [33]. Some antibiotics, specifically antibiotic growth promotors are known to lead to increased growth [34], yet in this case, although the bodyweight of restricted pigs became numerically-higher on days 18 and 21 following treatment, these effects were not significant.

### Microbial differences on day 42

On day 42, the day of the enriched group’s weaning, the two groups exhibited significant faecal microbiota differences, while no differences were observed in humoral immune responses, namely IgG levels on day 46. The overall composition of the faecal microbiota, as measured by β-diversity, was significantly different for both the Bray-Curtis and Sørensen metrics. In contrast, no significant differences were found in terms of α-diversity, likely owing to the diet. This would suggest that differences were a result of contrasting taxonomies, rather than the overall diversity of taxa. Although the enriched group were still suckling until day 42, they were also provided with the same solid feed as their restricted counterparts, hence increasing the complexity of dietary substrates, albeit to a lesser extent than the restricted pigs. Indeed, following the provision of solid feed, enriched piglets were observed to suckle readily and eat solid feed concomitantly, which has previously been shown to accelerate microbiota maturation in the form of increased α-diversity [35].

While limited to the genus level, LEfSe analysis highlighted genera such as *Escherichia-Shigella*,* Helicobacter* and *Streptococcus* as being associated with the enriched group in the faeces on day 42. While these genera would often be considered pathobionts, or even pathogens, they are frequently observed in the microbiota of healthy, younger piglets, particularly given that many are microaerophilic or facultatively anaerobic, and are thus better-suited to colonising the less-anaerobic, less-diverse intestinal milieu of younger animals [36, 37]. *Escherichia*,* Helicobacter* and *Streptococcus* are all characteristic of the microbiota during the piglet suckling phase [38–42]. In contrast, genera such as *Romboutsia* and the *Eubacterium coprostanoligenes* group, associated with the restricted piglets in the faeces on day 42, are known to ferment the plant-derived carbohydrates typical of a solid diet and produce SCFAs, and have hence been associated with older pigs [43–46]. *Dorea* proves somewhat more difficult to characterise, with some studies classifying it as associated with pre-weaning states [47, 48], and others associating it with post-weaning microbial changes [7, 41]. Caution should also be taken in overinterpreting individual taxa, given that many genera can have contrasting properties at the species or strain level. All in all, while there are some potentially detrimental genera associated with the enriched pigs, the differences at this stage may simply represent two relatively healthy microbiota types, just at different stages of maturation.

### Microbiota maturation across the two groups

Numerous studies have previously characterised the pre- and post-weaning microbiota of piglets. While taxa such as *Escherichia*,* Lactobacillus johnsonii*, and *Acinetobacter* frequently decrease following weaning, they are gradually replaced by *Megasphaera*, *Catenibacterium*, and *Prevotella copri*, and according to some studies, *Dorea* in the initial weeks after weaning [7, 44, 49]. While there exists some overlap between the established processes of gut microbiota maturation, namely increased *Escherichia* among enriched pigs, and increased *Dorea* among restricted pigs, our cohort likely presents a more complex picture of microbial maturation. Given that the enriched pigs consumed both solid feed and milk, this likely results in a microbiota that is in some regards taxonomically less mature compared to that of the restricted group, while being nonetheless no less diverse, and capable of degrading plant-derived fibres. This, alongside the bodyweight results with no stagnation in bodyweight gain following weaning, may suggest an advantage for the enriched group at weaning.

### Weaning age has no impact on serum IgG

What is more, weaning age was found to have no effect on serum IgG, as evidenced on days 11 and 46. Born agammaglobulinaemic, piglets rely upon the sow’s colostrum and milk in the early stages of life as their sole source of IgG [50, 51]. However, given that the gut barrier closes 24–36 h post farrowing [51], and immunoglobulin concentrations decrease over time in the sow’s milk [52], it is logical that the additional suckling period for enriched piglets conveyed no effects on serum IgG. These findings complement the microbiota composition and support the notion that the enriched microbiota was not pathogenic, or causing systemic immune responses, or IgG would have likely been elevated.

### Understanding the impact on the ileum by day 90

Ileal samples from day 90 permit further investigation into the longer-term, localised effects on the small intestine given the ileum’s key role as an interface between the microbiota and immune system. Limited differences however were observed in the microbiota itself. As a microoxic environment, the ileum hosts a less-diverse bacterial community compared to the faeces, or large-intestinal compartments [53–55], potentially explaining the reduced compositional differences. This decreased complexity of the ileal microbiota is reiterated in the consistently lower α-diversity values for the ileum, compared to both the day 42 and 90 faeces. *Enterococcus* alone was higher in the ilea of enriched pigs. *Enterococcus* is typically considered a component of the healthy microbiota, and can be utilised as a probiotic [56], although some species/strains can equally be pathogenic [57]. Conversely, *Limosilactobacillus*, unclassified Pasteurellaceae and *Escherichia* were characteristic of the restricted pigs’ ilea on day 90. While *Limosilactobacillus* is considered beneficial for pig gut health [58], *Escherichia* is often considered negative post-weaning [59], although some probiotic strains do exist [60]. Pasteurellaceae, commonly found in the ileal microbiota, has been associated with dietary protein [61].

Despite limited taxonomic differences, ileal histomorphology revealed distinct structural differences between the two groups on day 90. Increased weaning age has previously been associated with increased villus height and VH:CD ratios [62, 63], although some studies demonstrate contradictory results [64, 65]. In the present study, the enriched group exhibited significantly increased ileal VH:CD ratios, with a trend towards increased villus height. Likewise, both the VH:CD ratio and villus height were negatively correlated with serum IgG levels. That is to say, individuals with reduced villus heights were more likely to have undergone increased adaptive immune responses between days 42 and 90. Nevertheless, morphological changes were not replicated in changes in the gene expression of the gut barrier genes tested (*OCLN*,* CLDN1*, and *ZO1*). Future work investigating immune cell infiltration or inflammation scores would likely be interesting to better elucidate the exact nature of the changes observed herein.

### The faecal microbiota and VFAs exhibit differences by group on day 90

The faecal microbiota displayed marked differences between the two groups on day 90. As on day 42, β-diversity revealed distinct microbial compositions between the two groups for the Bray-Curtis and Sørensen indices. Enriched pigs also exhibited higher α-diversity for the Pielou evenness and Simpson indices, indicating that species evenness was higher in the enriched group, while species richness was unaffected. Previously, Wen et al. provided material including wood shavings, straw and peat as litter, but did not find any significant effects on α-diversity post-weaning, while Kubasova et al. found no effects on four-day-old piglets’ microbiotas after a short period of straw enrichment [13, 14]. Additionally, the VFA results indicate altered microbiota and gut functionality between the two groups. In the faeces, BCFAs as well as the SCFA butyrate were significantly higher among restricted pigs. While butyrate is well known to be produced via the fermentation of dietary fibre, it is also a product of protein fermentation [66], potentially explaining its elevated levels alongside BCFAs which are solely produced from proteins. The presence of BCFAs is sometimes considered negative for gut health considering their association with other harmful proteolytically-produced metabolites [66]. Nevertheless, recent studies analysing the impacts of specific BCFAs, namely isobutyrate, which was also elevated in the colon of restricted pigs, has revealed potentially beneficial roles against irritable bowel diseases and intestinal damage [67, 68].

### Understanding immunological differences between the groups

Both the spleen and blood help to provide a more systemic overview of immune responses beyond the gut itself. Firstly, the percentage of granulocytes and neutrophils was found to be greater in enriched pigs, suggesting a shift towards innate immune defences, exemplified further by the elevated N:L ratio. Nevertheless, neutrophil activation was higher in the restricted group, suggesting that enriched pigs had a greater proportion of their leucocytes dedicated to innate immunity, but were less activated, whereas restricted pigs had a reduced proportion of granulocytes/neutrophils, yet these were highly stimulated. These results are in contrast with Salak-Johnson and Webb’s study comparing day-14 and day-28-weaned piglets, where earlier weaning resulted in neutrophilia [69].

The cytokines IL-6, IL-15 and IFN-γ, the splenic gene expression of which was deemed by GSEA to be significantly higher in the restricted pigs, are well known for their involvement in inflammatory processes. IL-6 and IL-15 work synergistically to facilitate T-cell differentiation as well as IL-15 acting as an autocrine regulator to promote IL-6 production from macrophages [70, 71]. In accordance with the increased serum IgG among restricted pigs on day 90, IL-6 is a known growth factor for plasma cells which hence stimulates IgG production [72]. IL-15 is also known to activate the NF-κB transcription factor [73], which was also flagged as a core enrichment gene in restricted pigs by GSEA. IFN-γ is released by activated T cells, and drives antimicrobial responses [73]. Given that both IL-6 and IFN-γ are produced by lymphocytes, this may reflect the higher leucocyte counts among the restricted group. Additionally, IFN-γ induces the production of CXCL10, another of the core enrichment genes identified by GSEA, which in turn exerts pro-inflammatory effects through leucocyte recruitment and activation [74]. Additionally, CXCL13 increases during adaptive immune responses whereby it promotes the homing of naïve B cells into the follicles of the spleen [75]. The last chemokine identified by GSEA as significant, CCL2, is induced by inflammatory stimuli including IL-6 and IFN-γ, as well as bacterial molecules such as lipopolysaccharide, and has the primary function of a monocyte chemoattractant [76]. These gene expression data, alongside the serum IgG and leucocyte quantification, suggest that by day 90, the restricted pigs had mounted increased adaptive immune responses than those of the enriched group.

### Haematology

Haematology further revealed that a number of erythrocyte-related parameters were significantly different between the two groups, the reasons for which are likely multifactorial. Elevated MCV, a greater percentage of macrocytes, and a reduced percentage of microcytes all indicated that enriched pigs had, on average, larger erythrocytes than their restricted counterparts. Additionally, RDW-SD, a measure of anisocytosis, suggested that enriched pigs had a greater range of erythrocyte sizes. Similar to our findings, Wen et al. previously reported that the provision of bedding material was associated with elevated haemoglobin and MCV [14]. In our study, we presented several significant correlations of ileal and faecal bacteria with erythrocyte parameters. Murine models have shown that microbiota-derived lactate activates the secretion of stem cell factors from bone marrow LepR^+^ cells, thereby driving erythropoiesis [77]. While lactate was not measured in the current study, *Limosilactobacillus* (ileum), and *Fournierella* (faeces) are known lactate producers [78, 79], and proved positively correlated with MCHC in the present dataset. Erythropoiesis and haemoglobin synthesis have furthermore been demonstrated in vitro to be directly impaired by IL-6 and IFN-γ [80, 81], cytokines which GSEA associated with the restricted group. Moreover, erythrocyte size and volume is influenced by iron, and the B-group vitamins folate and vitamin B_12_ (cobalamin) [82]. As pigs are unable to synthesise these B-group vitamins endogenously, the gut microbiota is essential for their production [83]. The CD320 transcobalamin receptor facilitates cellular uptake of vitamin B_12_, with *CD320*-deficient mice known to exhibit megaloblastic anaemia [84]. In the present study, gene expression analysis in the spleen revealed upregulated *CD320* in restricted pigs, although this was not FDR-significant, so remains a possible explanatory mechanism, but is by no means definitive. Given that diet was identical for both groups, if there were systemically altered vitamin B_12_ levels, the gut microbiota would likely be the source. Likewise, the *FTH1* ferritin heavy chain 1 gene was upregulated in enriched pigs. Ferritin exhibits ferroxidase activity converting ferrous iron (Fe^2+^) to ferric (Fe^3+^) iron [85]. When physiological iron decreases, ferritin translation is downregulated leading to the release of iron. This would therefore reflect elevated iron levels in the bloodstream of the enriched pigs. Erythrocyte values were within normal physiological ranges for both groups, yet said differences between enriched and restricted pigs may indicate increased erythropoiesis in the enriched group, potentially due to increased lactate-producing bacteria, immune responses, or increased availability of iron or vitamin B_12_.

### The effect of litter of origin

Taken together, the present data indicate a significant link between rearing conditions in early life, and the piglets’ microbiota and physiology. Nevertheless, given the nature of the experimental conditions, the effect of litter of origin was poorly distinguishable from experimental group. The modelling performed however suggested a likely association of litter of origin with serum IgG and gut morphology on day 90. Although sows in the present study were controlled for parity and genetics per treatment group, individual differences cannot be eliminated entirely. Sow’s milk for instance is known to contain immunoglobulins, bioactive compounds, and prebiotic milk oligosaccharides, which, alongside its macronutrients can influence the physiology and microbiota of the piglet [86]. Curiously, the present data suggest no sow/litter effect on IgG on day 11, but effects on days 46 and 90. More broadly, nested PERMANOVA indicated a likely additional effect of litter of origin in influencing the microbiota’s composition alongside the experimental group. This is in line with previous research which has found small yet significant impacts of litter on the faecal microbiota [35]. Accordingly, litter-associated differences in the microbiota could be associated with such delayed physiological effects, although more research would be needed to confirm this.

### Potential implications for swine husbandry and porcine health

The present study sought not to reproduce current farming in its entirety, but instead to emulate two extreme ends of the husbandry spectrum. In contrast to previous studies, we opted for a combinatory treatment targeting three important variables of pig husbandry: weaning, litter material, and antibiotic use. A dual antibiotic treatment was administered in the first week of life; this is two weeks earlier than the common onset of post-weaning diarrhoea in industry, but sought to maximise early-life microbiota disruption and subsequent microbial succession. Early-life antibiotic administration had not yet been combined with such a prolonged weaning age, or the provision of litter material, factors which are equally important in the farming sector. Overall, restricted rearing resulted in a distinct faecal microbiota by day 42, the maturation of which was likely accelerated at weaning. As is often the case in the sector, weaning of the restricted group also resulted in a stagnation in bodyweight gain the following week. By day 90, the restricted group had reduced α-diversity compared to their enriched counterparts, and exhibited higher colonic and faecal BCFAs, the presence of which can be associated with both negative and positive health outcomes [66, 67]. Gene expression, as well as neutrophil activation parameters also indicated possible signs of inflammation, while leucocyte analysis indicated a higher percentage of lymphocytes. The enriched group on the other hand, had faecal microbiotas likely adapted towards both a milk- and plant-derived diet on day 42, and saw no stagnation in bodyweight gain the following week. Although the N:L ratio, raised in enriched pigs on day 90, is frequently used as a proxy for inflammation [87], this may reflect a greater percentage of neutrophils which were less activated than in the restricted group. Erythrocyte parameters were also altered by rearing conditions, with the enriched group exhibiting signs of increased erythropoiesis.

Taken together, it remains difficult to establish whether one rearing system produced truly ‘healthier’ animals than the other, despite clear differences. It should also be noted that, as conducted on a controlled research farm, this setup may not reflect the entirety of variation seen in the real world. A greater number of animals, across a greater number of litters would also be helpful in future work to consolidate findings. Nevertheless, we demonstrate that enriched rearing likely had benefits over restricted conditions based on the likely adaptation of the day 42 faecal microbiota to weaning, and the resultant increased faecal α-diversity by day 90. While likely also impacted by the litter of origin, enriched rearing was also associated with higher VH:CD in the ileum, and downregulation of pro-inflammatory genes in the spleen. As such, in industry, it is important to be mindful that the aspects of enriched rearing: later weaning, bedding material, and no antibiotics may have positive implications for porcine health in the long run.

## Conclusions

Previous studies have evaluated the impacts of different rearing conditions and early-life interventions, drawing mixed conclusions regarding the nature and longevity of their effects. The present study sought to combine said factors into a single model, thereby simulating ‘restricted’ and ‘enriched’ rearing conditions, and exploring their long-term impacts until day 90. We demonstrated that these two rearing conditions led to distinct microbiota profiles, with enriched husbandry resulting in improved gut morphology, and the restricted group presenting with elevated colonic and faecal BCFAs, indications of inflammation via gene expression, and increased serum IgG. This work demonstrates that changes in early-life factors can indeed lead to significant physiological and immunological consequences later in life.

## Supplementary Information

Below is the link to the electronic supplementary material.

Supplementary Material 1

Supplementary Material 2

## Data Availability

Raw sequencing data are accessible as FASTQ files via the NCBI platform, publicly available from 1 May 2025. Accession number: PRJNA1253086.

## Abbreviations

BCFA: Branched-chain fatty acid
GSEA: Gene set enrichment analysis
HO-1: Haem oxygenase 1
IgG: Immunoglobulin G
LEfSe: Linear discriminant analysis effect size
MCH: Mean corpuscular haemoglobin
MCHC: Mean corpuscular haemoglobin concentration
MCV: Mean corpuscular volume
N:L: Neutrophil to lymphocyte (ratio)
PCA: Principal component analysis
PCoA: Principal coordinate analysis
PRR: Pattern recognition receptor
qPCR: Quantitative PCR
RDW-SD: Red cell distribution width – standard deviation
SCF-1: Specimen collection fluid-1
SCFA: Short-chain fatty acid
SEM: Standard error of the mean
VFA: Volatile fatty acid
VH:CD: Villus height to crypt depth (ratio)
