# Supplementary material for: The impact of early-life rearing conditions on the porcine gut microbiota and immune system
Source: Anim Microbiome. 2025 Dec 5;7:125. doi: 10.1186/s42523-025-00492-y (PMC12681121; doi:10.1186/s42523-025-00492-y)
Supplement: Supplementary file 1 — Supplementary Material 1 [file 42523_2025_492_MOESM1_ESM.docx]

| **Category** | **Gene** | **Full name** | **Description** | **Primers (5’ → 3’)** | **Accession no.** | **Reference** |
| --- | --- | --- | --- | --- | --- | --- |
| Housekeeping | *B2M* | Beta-2-microglobulin | β-2-microglobulin | F: CAAGATAGTTAAGTGGGATCG  R:TGGTAACATCAATACGATTTC | XM_021096362.1 | [1] |
|  | *HMBS* | Hydroxymethylbilane synthase |  | F: AGGATGGGCAACTCTACCTG  R: GATGGTGGCCTGCATAGTCT | XM_021102148.1 | [1] |
|  | *HPRT1* | Hypoxanthine phosphoribosyltransferase 1 |  | F: GGACTTGAATCATGTTTGTG  R: CAGATGTTTCCAAACTCAAC | XM_021079504.1 | [1] |
|  | *PGK1* | Phosphoglycerate kinase 1 |  | F: GGGCTAAGCAGATTGTATG  R: TGACTTTATCCTCCGTGTT | NM_001099932.2 | [1] |
|  | *HSPCB* | Heat shock protein 90 alpha family class B member 1 |  | F: GGCAGAAGACAAGGAGAAC  R: CAGACTGGGAGGTATGGTAG | XM_005666063 | [1] |
|  | *ACTB* | Actin beta | β-actin | F: CTACGTCGCCCTGGACTTC  R: GCAGCTCGTAGCTCTTCTCC | XM_021086047.1 | [2] |
|  | *GAPDH* | Glyceraldehyde-3-phosphate dehydrogenase |  | F: GTCGGAGTGAACGGATTTGGC  R: GGAGGTCAATGAAGGGGTCA | NM_001206359.1 | *De novo* |
|  | *ESPN* | Espin |  | F: TCCACTGGCAGCACCAAG  R: CAGACTCTTGCCTGCCTTGA | XM_003127530.4 | *De novo* |
|  | *RPL13A* | Ribosomal protein L13a |  | F: GGAGGGGCAGCAGGTG  R: TGCCAGAAATGTTGATGCCC | NM_001244068.1 | *De novo* |
|  | *RPL32* | Ribosomal protein L32 |  | F: GCTGCTAATGTGCAACAAATCATA  R: GCCGCTCTCTCGACGAT | XM_021068582.1 | *De novo* |
|  | *RPL4* | Ribosomal protein L4 |  | F: AGGTTTATGCCTCTCAGCGAA  R: GTTAAGCAGAGTAATTCCAGGGATG | XM_005659862.3 | *De novo* |
|  | *YWHAZ* | Tyrosine 3-monooxygenase/tryptophan 5-monooxygenase activation protein zeta |  | F: GAACTCCCCAGAGAAAGCCT  R: TCCGATGTCCACAATGTCAAG | NM_001315726.1 | *De novo* |
|  | *TBP* | TATA-box binding protein |  | F: AGTTCTGTAGGGTCGGGAAGG  R: ACAGCAAGAAAGAGTGATGCTGGA | XM_021085483.1 | *De novo* |
| Cytokines | *IFNA1* | Interferon alpha 1 | IFN-α1 | F: AGAAGCATCTGCAAGGTTCC  R: GATGGCATTGCAGCTGAGTAG | NM_214393.1 | *De novo* |
|  | *IFNB1* | Interferon beta 1 | IFN-β1 | F: GTTGCCTGGGACTCCTCAAT  R: ACGGTTTCATTCCAGCCAGT | NM_001003923.1 | *De novo* |
|  | *IFNG* | Interferon gamma | IFN-γ | F: TGAAGAATTGGAAAGAGGAGAGTGA  R: GCTCCTTTGAATGGCCTGGT | NM_213948.1 | *De novo* |
|  | *IFNL3* | Interferon lambda 3 | IFN-λ3 | F: GAGGCCCAGAAGAAGGAGC  R: TGTCTGGGGTCAGACACAC | XM_021097830.1 | *De novo* |
|  | *IFNW1* | Interferon omega 1 | IFN-ω1 | F: GCATCTGGAAGACCTGGAGTC  R: CATGATTTCCACTCTGACAATCTCC | NM_001130238.1 | *De novo* |
|  | *IL1B* | Interleukin 1 beta | IL-1β | F: GCCAGTCTTCATTGTTCAGGTTT  R: ATCTCTTTGGGGCCATCAGC | NM_214055.1 | *De novo* |
|  | *IL4R* | Interleukin 4 receptor | IL-4 receptor | F: GTGGCCCATCTGCCTATCC  R: CTGAGCCTGCTCTGTTCTCG | NM_214340.1 | [3] |
|  | *IL6* | Interleukin 6 | IL-6 | F: CCCTGAGGCAAAAGGGAAAGA  R: AGGAAATCCTCAAGGCTGCG | NM_214399.1 | *De novo* |
|  | *IL8* | Interleukin 8 | IL-8 | F: TGGACCCCAAGGAAAAGTGG  R: TGTTGTTGCTTCTCAGTTCTCT | NM_213867.1 | *De novo* |
|  | *IL10* | Interleukin 10 | IL-10 | F: CGGCCCAGTGAAGAGTTTCT  R: GGCAACCCAGGTAACCCTTA | NM_214041.1 | *De novo* |
|  | *IL12B* | Interleukin 12B | IL-12p40 | F: TCGTGCTGGAAGCTGTTCAC  R: GGAGGGTCTGGTTTGATGATGT | NM_214013.1 | *De novo* |
|  | *IL15* | Interleukin 15 | IL-15 | F: TGCATCCAGTGCTACTTGTGT  R: CCTGCACTGATACAGCCCAA | XM_021100480.1 | *De novo* |
|  | *IL17A* | Interleukin 17A | IL-17A | F: GGACAAGAACTTCCCTCAGCA  R: TCCTCGTTGCGTTGGAGAG | NM_001005729.1 | *De novo* |
|  | *IL18* | Interleukin 18 | IL-18 | F: AGCTGAAAACGATGAAGACCT  R: CAAACACGGCTTGATGTCCC | NM_213997.1 | *De novo* |
|  | *IL22* | Interleukin 22 | IL-22 | F: AAGCTAAGCCAATGCCGTAT  R: TTGTCCCTTCTAAGATCAGGTGAG | XM_021091967.1 | *De novo* |
|  | *IL33* | Interleukin 33 | IL-33 | F: ACCAGATCACAAGAAGCCTG  R: GCCGGCTGAGCTATTCATCT | NM_001285978.1 | *De novo* |
|  | *EBI3* | Epstein-Barr virus induced 3 | IL-27β, IL-35β | F: CCGTGGGAGAGAAGCAGC  R: AGCCTGTACGTGGCAATGAA | NM_001315682.1 | *De novo* |
|  | *TNFA* | Tumour necrosis factor alpha | TNF-α | F: TCTGCCTACTGCACTTCGAG  R: GTTGATGCTCAAGGGGCCA | NM_214022.1 | [2] |
|  | *TGFB1* | Transforming growth factor beta 1 | TGF-β1 | F: CTGGAAAGCGGCAACCAAAT  R: GCCCGAGAGAGCAATACAGG | XM_021093503.1 | *De novo* |
| Chemokines | *CCL2* | C-C motif chemokine ligand 2 |  | F: TCCCACACCGAAGCTTGAATC  R: TGGAGAATTAATTGCATCTGGCTG | NM_214214.1 | *De novo* |
|  | *CCL5* | C-C motif chemokine ligand 5 |  | F: CTCCATGGCAGCAGTCGT  R: AAGGCTTCCTCCATCCTAGC | AJ583704 | [4] |
|  | *CCR7* | C-C motif chemokine receptor 7 |  | F: GAGTGACATGGACCTGGGGAA  R: ATGTAGTCGTCCGTGACCTC | NM_001001532.3 | *De novo* |
|  | *CXCL8* | C-X-C motif chemokine ligand 8 |  | F: AGAGTGGACCCCACTGTGAA  R: TGTTGTTGCTTCTCAGTTCTCTT | NM_213867.1 | *De novo* |
|  | *CXCL10* | C-X-C motif chemokine ligand 10 |  | F: CCCACATGTTGAGATCATTGC  R: GCTTCTCTCTGTGTTCGAGGA | NM_001008691.1 | [2] |
| Signalling pathways | *AKT1* | AKT serine/threonine kinase 1 |  | F: CTGCACAAACGAGGCGAGTA  R: ATGAAGGTGCCGTCGTTCTT | NM_001159776.1 | *De novo* |
|  | *MAPK14* | Mitogen-activated protein kinase 14 |  | F: TTAAGACTCGTTGGAACCCCAG  R: GCAAGTCAACAGCCAAGGGA | XM_001929490.6 | *De novo* |
|  | *MYD88* | MYD88 innate immune signal transduction adaptor |  | F: GGCCCAGCATTGAAGAGGAC  R: TGACATCCAAGGGATGCTGC | NM_001099923.1 | *De novo* |
|  | *NFKB1* | Nuclear factor kappa B subunit 1 | NF-κB p50 subunit | F: GGACAACTACGAGGTCTCCG  R: GCCTGAGAGGTGGTCTTCAC | NM_001048232.1 | *De novo* |
|  | *NFKBIA* | NFKB inhibitor alpha | IκBα | F: ACCAACTACAATGGCCACAC  R: GCACCCAAAGACACCAACAG | NM_001005150.1 | *De novo* |
|  | *GATA3* | GATA binding protein 3 |  | F: GAGGTCCAGCACAGAAGGCA  R: AAGGGGTCGATTCTGTCCGT | XM_021064072.1 | *De novo* |
|  | *COX2* | Cytochrome c oxidase subunit II |  | F: GCAGGCTGATACTGATAGGAGA  R: TGGTAGCCACTCAGGTGTTG | NM_214321.1 | *De novo* |
|  | *ISG15* | ISG15 ubiquitin like modifier |  | F: CAGAGCGCACAGAGGCAG  R: GTTCCCTACCCATGGCCG | NM_001128469.3 | *De novo* |
| Immunoglobulin | *IGHM* | Immunoglobulin heavy constant mu | IgM (µ chain) | F: AGACCCTACAGTCCAGTCCG  R: GAGGACGCGTTCTTCTCGTA | ENSSSCT00000062470.3 | *De novo* |
|  | *IGHA* | Immunoglobulin heavy constant alpha | IgA (α chain) | F: CAGATCCATGTCCCCAGTGC  R: GAAGCTGACACCCTCGGATT | U12594.1 | *De novo* |
|  | *IGHE* | Immunoglobulin heavy constant epsilon | IgE (ε chain) | F: ACCTGTACGTCCACAAGTCG  R: CCTGGACCACAACAGGTTCA | ENSSSCT00000099686.1; ENSSSCT00000064831.3 | *De novo* |
| Cell markers | *MPO* | Myeloperoxidase | Neutrophil enzyme | F: CCCGAGTTGCTTTCCTCACT  R: AAGAAGGGGATGCAGTCACG | XM_003131655.3 | [5] |
|  | *FOXP3* | Forkhead box P3 | Marker of regulatory T cells | F: AACAGCACGTTCCCAGATTTC  R: GATCTCGTTGAGTGTCCGCT | XM_021079538.1; XM_021079539.1; XM_021079537.1; NM_001128438.1 | *De novo* |
|  | *CD4* | Cluster of differentiation 4 | Marker of CD4^+^ T cells | F: TTGCTGGTGTTCAGACTGACT  R: TTGCACTGTAGGGTGACTCC | XM_021091199.1; XM_021091198.1; XM_005652590.3; NM_001001908.2 | *De novo* |
|  | *CD8A* | Cluster of differentiation 8 subunit alpha | Marker of CD8^+^ T cells | F: CCAGCCCTTCAGAGAGATTCA  R: CTGGGGAAACGGAAAGGAAAG | XM_021085821.1 | Zhang |
|  | *CTLA4* | Cytotoxic T-lymphocyte associated protein 4 | Marker of activated T cells/Tregs | F: CCCTGTCTTCTCCAAAGGGAT  R: TTGCCTGCAGACCCATACTC | NM_214149.1 | *De novo* |
|  | *CD163* | Cluster of differentiation 163 | Marker of macrophages | F: AGCGGCTCTCAGTTTTCTCA  R: AGAGTGGTCTCCTGAGGGATT | NM_213976.1 | *De novo* |
|  | *CD83* | Cluster of differentiation 83 | Marker of mature dendritic cells (and Tregs) | F: ACTCATCATTTTCACCTGCAAGTTT  R: CACTGGCTCCAAGGGCTTAT | XM_001928655.5 | *De novo* |
|  | *CD205* | Cluster of differentiation 205 | Marker of dendritic cells and monocytes | F: CTGGCCACGCAGATAAGGAT  R: CCAGTCGTTCAGTGGTGTGA | NM_001184946.1 | *De novo* |
|  | *ITGAM* | Integrin subunit alpha M | Marker of neutrophil activation | F: CTTTGTGCCTCCTACTTCTCT  R: TGACTTAAGGCCAAGGCTGT | XM_003124492.6 | *De novo* |
|  | *CR2* | Complement C3b/C4b receptor 1 | Complement receptor (marginal zone B celsl) | F: GCCTTTTTGGCTTCACCATGA  R: AGGAGGTGCTTGACAATCCTT | XM_021063334.1 | *De novo* |
|  | *CCR3* | C-C motif chemokine receptor 3 | Marker of eosinophils and basophils | F: CTATGGGGACTGGTCAAGGG  R: GGTTTCCATTTTTCTTGTCCCTGT | NM_001001620.1 | *De novo* |
|  | *SDC1* | Syndecan 1 | Marker of plasma cells | F: CCAGCTTGACCCTCACACTC  R: GTCAAAGGTGAAGTCCGGCTC | NM_001243190.1 | *De novo* |
|  | *NCR1* | Natural cytotoxicity triggering receptor 1 | Marker of NK cells | F: GTGAAGCTCCTGGTCAAAGGA  R: TCCCAGAGGTCAGGCTCTTTC | XM_005664831.3; NM_001123143.1 | *De novo* |
|  | *AREG* | Amphiregulin | Produced by ILC2s and Tregs | F: GATCCTCTGCTCAGCCCATT  R: CCTCACTTCCCGAGGACATC | NM_214376.1 | *De novo* |
|  | *SLA-DOA* | Major histocompatibility complex, class II, DO alpha |  | F: CTCTTTACGCACTGGGAGCC  R: GGCGTCTGCCTGTGATGATA | NM_001185143.1 | *De novo* |
| PRRs | *TLR1* | Toll-like receptor 1 |  | F: ACAGGTCATCTTGCCTTCAC  R: AATGACGCCTCGGTGATCC | NM_001031775.1 | *De novo* |
|  | *TLR2* | Toll-like receptor 2 |  | F: cggaagataatgaacaccaggac  R: atcgcagctctcaaatttaacca | XM_005653577.3 | [6] |
|  | *TLR4* | Toll-like receptor 3 |  | F: gtgctggatttatccagatgtga  R: gatttcccgtcagtatcaaggtg | NM_001113039.2 | [6] |
|  | *TLR5* | Toll-like receptor 5 |  | F: tcatgggtttatcttctccctga  R: gcttggtctgcaatcttgtttatc | NM_001348771.1 | [6] |
|  | *TLR6* | Toll-like receptor 6 |  | F: ccaaaagacctgccaccccaaacca  R: accgtcagctgcgagagaaagctg | NM_213760.2 | [6] |
|  | *TLR9* | Toll-like receptor 9 |  | F: ctcagaggacttcatgccaaact  R: actggattgtcaccaggttgttc | XM_005669564.3 | [6] |
|  | *MD2* | Lymphocyte antigen 96 |  | F: TTTTGCGGAGCTCTGAAGGG  R: ACACAATGGTATTGTCCCGGAG | NM_001104956.1 | *De novo* |
|  | *NOD2* | Nucleotide-binding oligomerisation domain containing 2 |  | F: gagcgcatcctcttaactttc  R: acgctcgtgatccgtgaac | NM_001105295.1 | [6] |
|  | *CLEC7A* | C-type lectin domain family 7 member A |  | F: tcaaggcatgtgtcttcccaacctga  R: ctcccaaagccatggcccttcagtc | XM_005655671.3 | [6] |
|  | *NLRP3* | NLR family pyrin domain containing 3 |  | F: TTTGGCTGTTCCTGAGGCAG  R: AGGGCATAGGTCCACACAAA | NM_001256770.2 | *De novo* |
| Haemoglobin/  iron | *HBA* | Haemoglobin subunit alpha |  | F: TCCCGACCCAGACTCAGAAA  R: AGCCCAGGAACATTCTTTCCA | XM_021086966.1 | *De novo* |
|  | *HBB* | Haemoglobin subunit beta |  | F: GCTTCTGACACAACCGTGTTC  R: CAACCAGCAGCCTGCCC | NM_001144841.1 | *De novo* |
|  | *LOC100515788* |  | Haemoglobin β-like subunits (ε/γ/δ) | F: ATCCTGAGAACTTCAGGCTCC  R: TACTAGTGGGCCAGGGCATT | *LOC100515788* | *De novo* |
|  | *TFRC* | Transferrin receptor |  | F: GAGGGCGGGTTCTTTTGTGT  R: GGTTCTCCGCCAAACAAACT | NM_214001.1 | *De novo* |
|  | *FTH1* | Ferritin heavy chain 1 |  | F: CCATGGAATTTGCGCTGCAC  R: TGAAGTCACACAAGTGGGGG | NM_213975.1 | *De novo* |
|  | *HMOX1* | Haem oxygenase 1 |  | F: GGCTGAGAATGCCGAGTTCA  R: GGACGCCATCACCAGCTTAAA | NM_001004027.1 | *De novo* |
|  | *SLC40A1* | Solute carrier family 40 member 1 | Ferroportin | F: AGGGATTGGATTGTTGTTGTTGC  R: TTCGTATTGTAGCATTCATATCTGC | XM_003483701.4 | *De novo* |
|  | *HAMP* | Hepcidin antimicrobial peptide |  | F: GTTCTCCCATCCCAGACAAGACA  R: GTGGGTGTCTCTTCTTAGCCT | NM_214117.1 | *De novo* |
|  | *CP* | Ceruloplasmin |  | F: CCAATACCAGCACAGGGGCATTTATAC  R: GCAGTGGAGTAACCAGGTTCCAG | XM_021068574.1 | [7] |
| Folate | *SLC19A1* | Solute carrier family 19 member 1 | Folate transporter 1 | F: CCGGCTACTACCTGATCGTC  R: AGGAAGTGATGGCACCGAG | XM_021071539.1 | *De novo* |
|  | *SLC46A1* | Solute carrier family 46 member 1 | Proton-coupled folate transporter | F: CTCATGTTCACAGGGTACGGG  R: ACCTTGCTCAGACACGCTC | NM_001243383.2 | *De novo* |
|  | *MTHFR* | Methylenetetrahydrofolate reductase | Folate cycle enzyme | F: TGTTTCCGGCGTCCACTG  R: CCATGGCTGAGCTCCTGTTAAT | XM_003127574.4 | *De novo* |
| Vitamin B_12_ (cobalamin) | *TCN2* | Transcobalamin 2 |  | F: GAAGGCCTAGAGGCAGCG  R: TCAGACAGGCCGGTTTTCTC | NM_001244436.1 | *De novo* |
|  | *CD320* | Cluster of differentiation 320 | Transcobalamin receptor | F: GTGATGAGGTGGAATGCGGTAT  R: GTGCCGGCAGTTCTCATTAAC | XM_021085137.1 | *De novo* |
|  | *MTR* | 5-methyltetrahydrofolate-homocysteine methyltransferase |  | F: CAGACCCCCTCTGCATTGGA  R: GGATAACAGAGGACATAGGCCG | XM_001927058.4 | *De novo* |
| Caspase | *CASP1* | Caspase 1 |  | F: CGAACTCTCCACAGGTTCAC  R: AAGACGCAGGCTTAACTGGG | NM_214162.1 | *De novo* |
|  | *CASP3* | Caspase 3 |  | F: AAGCAAATCAATGGACTCTGGAA  R: TTGCAGCATCCACATCTGTACC | NM_214131.1 | [8] |
| Sexing verification | *EIF1AY* | Eukaryotic translation initiation factor 1A, Y-linked | Y chromosome gene | F: GGAGCTTCCAGAACATGCAAA  R: TCCTCAGAAACTCTTGTAAGACCA | NM_001244349.1 | *De novo* |
|  | *EIF2S3Y* | Eukaryotic translation initiation factor 2 subunit 3 | Y chromosome gene | F: TGAATCAGAAAAGTATGGTGGGC  R: GTGTTCCGCAAAAAGTGAGACA | XM_021080929.1 | *De novo* |
| Gut integrity | *OCLN* | Occludin |  | F: CAGGTGCACCCTCCAGATTG  R: TATGTCGTTGCTGGGTGCAT | NM_001163647.2 | [9] |
|  | *CLDN1* | Claudin 1 |  | F: TCTTTCTTATTTCAGGTCTGGCT  R: ACTGGGGTCATGGGGTCATA | NM_001244539.1 | *De novo* |
|  | *ZO1* | Tight junction protein ZO-1 |  | F: CACCTTTAGATAAGGAGAAAGGTGA  R: ATCACAGTGTGGTAAGCGCA | XM_003480423.4 | *De novo* |
|  | *S100A8* | S100 calcium-binding protein A8 | Calprotectin | F: CTGACGGATCTGGAGAGTGC  R: GGTTTCTGCGTCCTTTTTCTTCA | NM_001160271.3 | *De novo* |

1. Wang S, Wang B, He H, Sun A, Guo C. A new set of reference housekeeping genes for the normalization RT-qPCR data from the intestine of piglets during weaning. PLoS One. 2018;13.

2. Uerlings J, Schroyen M, Bautil A, Courtin C, Richel A, Sureda EA, et al. In vitro prebiotic potential of agricultural by-products on intestinal fermentation, gut barrier and inflammatory status of piglets. British Journal of Nutrition. 2020;123:293–307.

3. Qi M, Tan B, Wang J, Li J, Liao S, Yan J, et al. Small intestinal transcriptome analysis revealed changes of genes involved in nutrition metabolism and immune responses in growth retardation piglets. J Anim Sci. 2019;97:3795–808.

4. Rødgaard T, Skovgaard K, Stagsted J, Heegaard PMH. Expression of innate immune response genes in liver and three types of adipose tissue in cloned pigs. Cell Reprogram. 2012;14:407–17.

5. Bæk O, Skadborg K, Muk T, Amdi C, Heegaard PMH, Thymann T, et al. Infant formula based on milk fat affects immune development in both normal birthweight and fetal growth restricted neonatal piglets. Nutrients. 2021;13.

6. Osvaldova A, Stepanova H, Faldyna M, Matiasovic J. Gene expression values of pattern-recognition receptors in porcine leukocytes and their response to Salmonella enterica serovar Typhimurium infection. Res Vet Sci. 2017;114:31–5.

7. Wilkinson JM, Gunvaldsen RE, Detmer SE, Dyck MK, Dixon WT, Foxcroft GR, et al. Transcriptomic and epigenetic profiling of the lung of influenza-infected pigs: A comparison of different birth weight and susceptibility groups. PLoS One. 2015;10.

8. Van Le Thanh B, Lemay M, Bastien A, Lapointe J, Lessard M, Chorfi Y, et al. The potential effects of antioxidant feed additives in mitigating the adverse effects of corn naturally contaminated with Fusarium mycotoxins on antioxidant systems in the intestinal mucosa, plasma, and liver in weaned pigs. Mycotoxin Res. 2016;32:99–116.

9. Chen Y, Xie Y, Zhong R, Han H, Liu L, Chen L, et al. Effects of graded levels of xylo-oligosaccharides on growth performance, serum parameters, intestinal morphology, and intestinal barrier function in weaned piglets. J Anim Sci. 2021;99.
