# Supplementary material for: The impact of early-life rearing conditions on the porcine gut microbiota and immune system
Source: Anim Microbiome. 2025 Dec 5;7:125. doi: 10.1186/s42523-025-00492-y (PMC12681121; doi:10.1186/s42523-025-00492-y)
Supplement: Supplementary file 2 — Supplementary Material 2 [file 42523_2025_492_MOESM2_ESM.docx]

**Supplementary Materials**

| **Component (%)** | **SUCKLING PHASE 1** | **SUCKLING PHASE 2** | **POST-WEANING PHASE 1** | **POST-WEANING PHASE 2** |
| --- | --- | --- | --- | --- |
| **Dry matter** | 92.00 | 90.50 | 89.50 | 88.72 |
|  |  |  |  |  |
| **Energy (MJ/kg)** | 11.88 | 10.86 | 8.77 | 9.44 |
|  |  |  |  |  |
| **Crude protein** | 19.00 | 17.00 | 16.80 | 17.20 |
| **Crude fat** | 10.00 | 7.00 | 4.40 | 4.50 |
| **Crude fibre** | 2.00 | 2.50 | 3.50 | 4.20 |
|  |  |  |  |  |
| **Lysine** | 1.40 | 1.15 | 1.21 | 1.20 |
| **Methionine** | 0.45 | 0.98 | 0.46 | 0.42 |

**Table S1. Dietary nutritional composition throughout the course of the experiment.**

Suckling phase 1 feed was provided from day 14 to 21. Between days 21 and 28, a 50:50 mixture of suckling phase 1 and 2 was provided. Suckling phase 2 feed alone was provided from day 28 to 35. Post-weaning phase 1 feed was provided from day 35 to 49. Post-weaning phase 2 feed was provided from day 49 onwards.

*(Table in separate document with extra references)*

**Table S2. Primers used for qPCR in the present study.**

| **Day** | **Restricted** | | **Enriched** | | ***P*-Value** |
| --- | --- | --- | --- | --- | --- |
|  | **Mean BW (kg)** | **SEM** | **Mean BW (kg)** | **SEM** |  |
| **0** | 1.34 | 0.03 | 1.40 | 0.03 | 0.211 |
| **3** | 1.91 | 0.05 | 1.93 | 0.05 | 0.823 |
| **11** | 3.52 | 0.10 | 3.43 | 0.11 | 0.586 |
| **18** | 4.94 | 0.16 | 4.75 | 0.16 | 0.425 |
| **21** | 5.65 | 0.23 | 5.32 | 0.19 | 0.271 |
| **27** | 5.77 | 0.23 | 6.48 | 0.23 | **0.041** |
| **42** | 9.72 | 0.37 | 10.30 | 0.38 | 0.282 |
| **49** | 11.92 | 0.42 | 12.48 | 0.45 | 0.363 |
| **69** | 21.46 | 0.70 | 23.53 | 0.93 | 0.080 |
| **90** | 37.68 | 0.79 | 40.71 | 1.26 | **0.044** |

**Table S3. Mean bodyweight (BW) by group from farrowing (day 0) to the endpoint of the experiment (day 90).**

Bodyweight is shown as the mean per group ± the standard error of the mean. The Wilcoxon test was performed to calculate the *P*-value. *N* = 64.


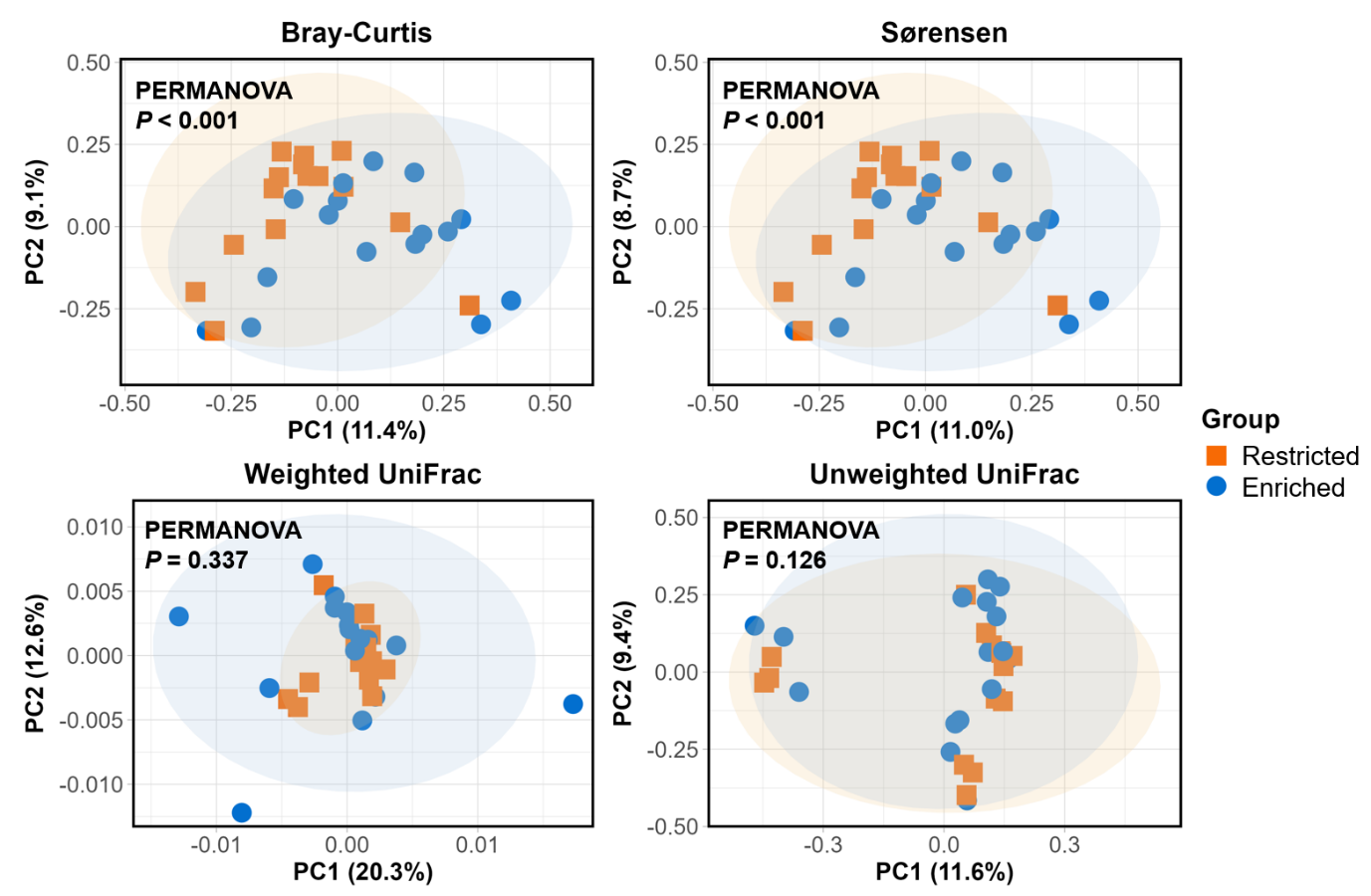


**Figure S1. β-diversity of the faecal microbiota on day 42.**

The graphs display β-diversity calculated according to Bray-Curtis, Sørensen, and weighted and unweighted UniFrac distances, with PERMANOVA indicating significant differences between groups at both timepoints (*P* < 0.01).


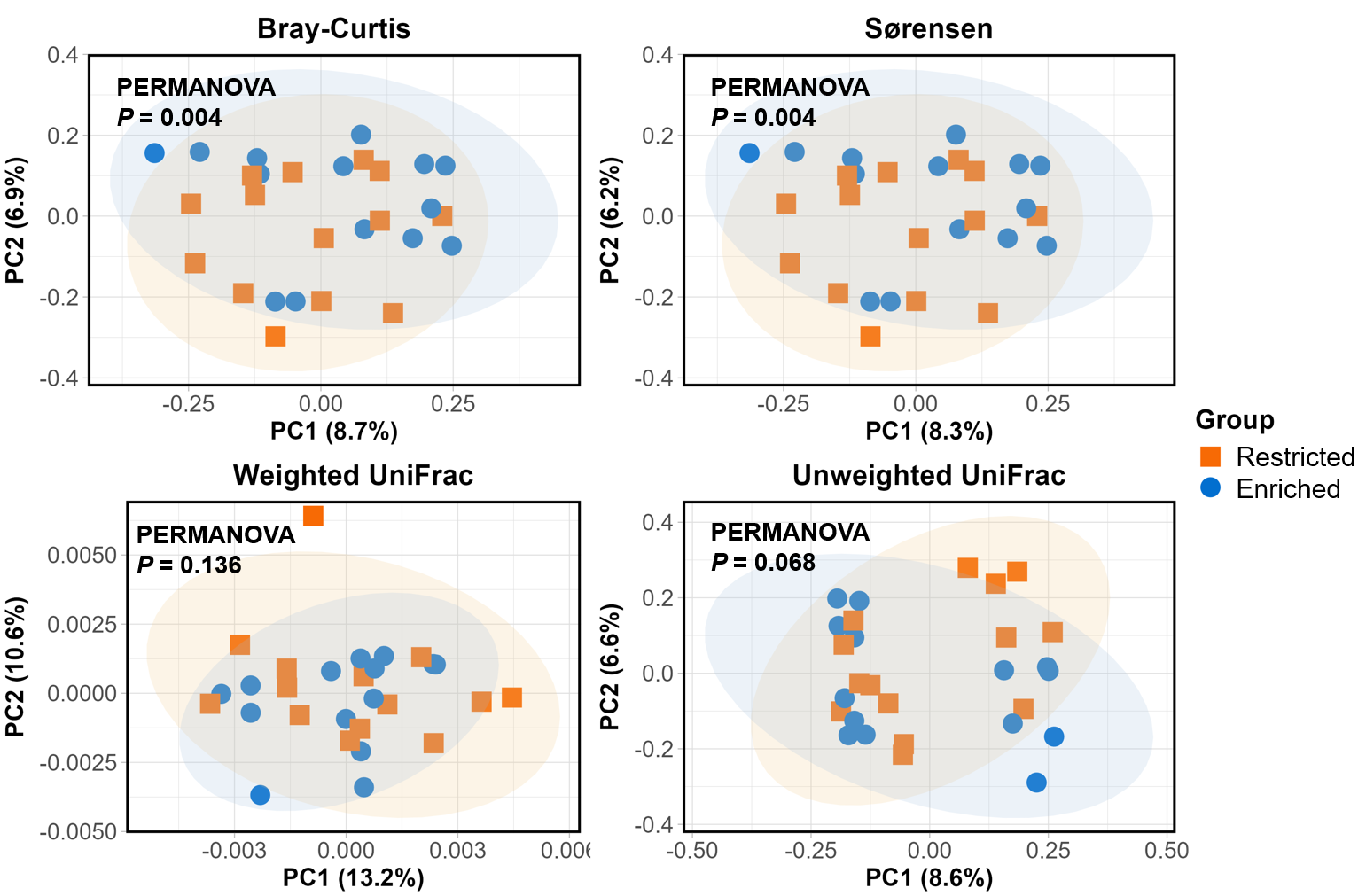


**Figure S2. β-diversity of the faecal microbiota on day 90.**

The graphs display β-diversity calculated according to Bray-Curtis, Sørensen, and weighted and unweighted UniFrac distances, with PERMANOVA indicating significant differences between groups at both timepoints (*P* < 0.01).


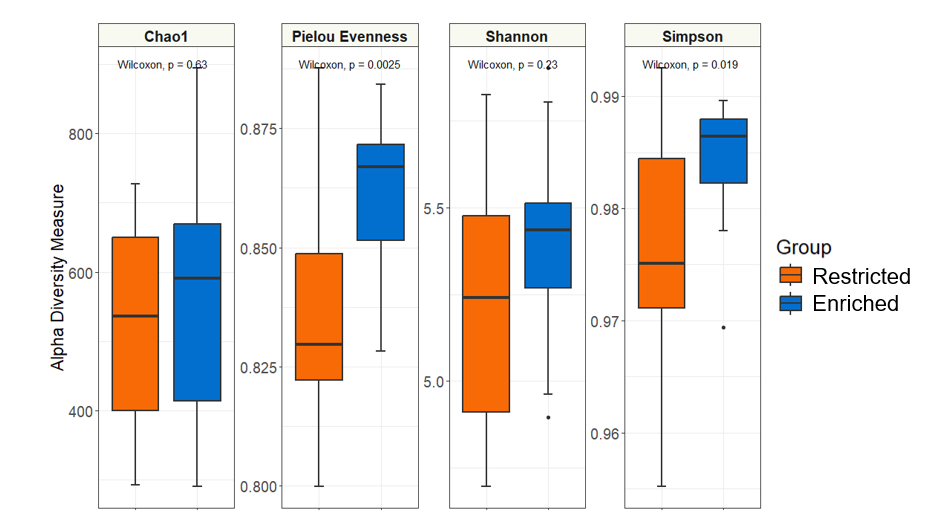


**Figure S3. α-diversity of the faecal microbiota on day 90.**

α-diversity is shown according to the Chao1, Pielou evenness, Shannon, and Simpson indices. Statistically significant differences were calculated by performing the Wilcoxon test.


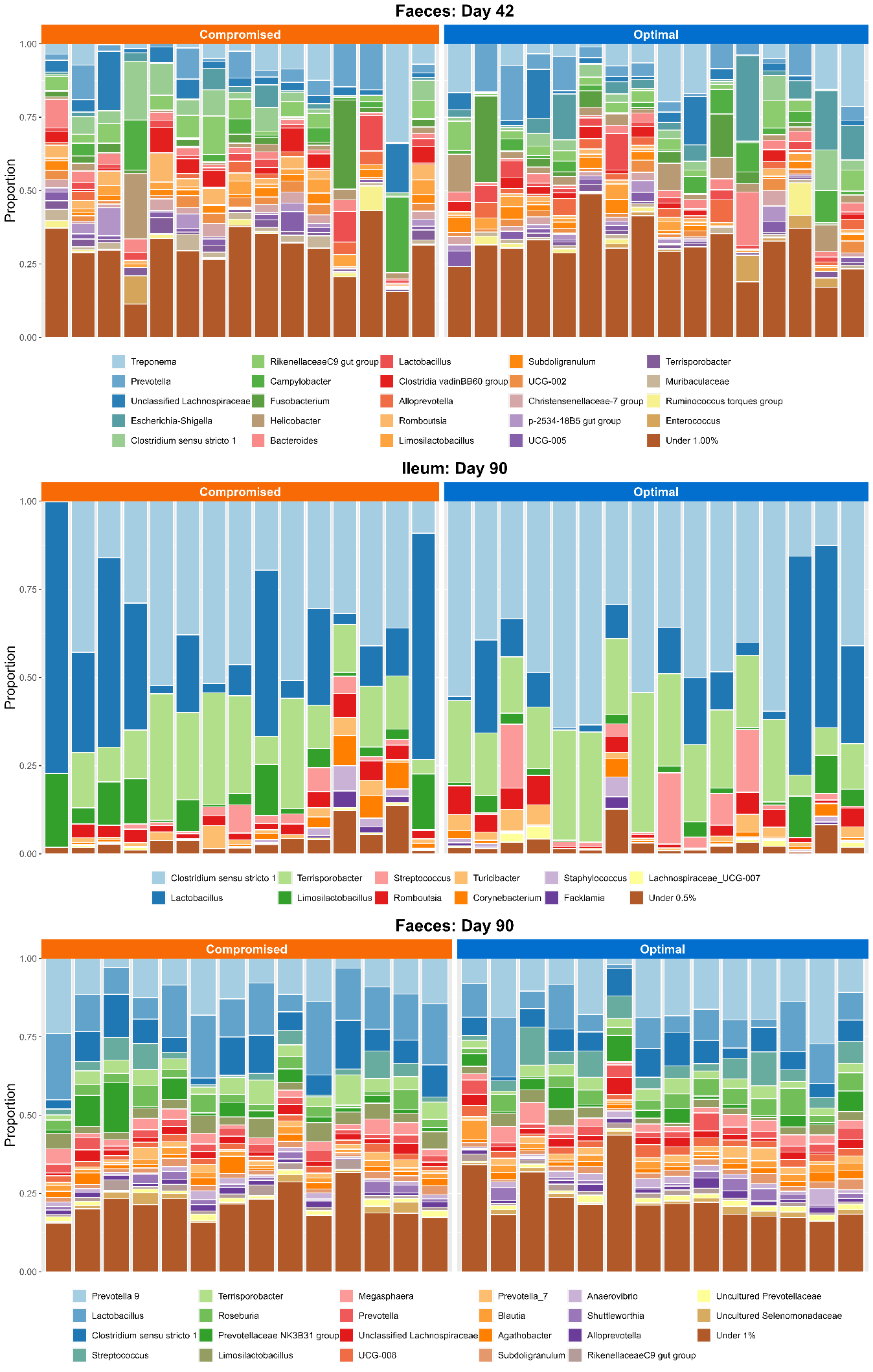
**Figure S4. Taxonomic abundance at the genus level for faeces day 42, ileum day 90 and faeces day 90**

**C**

**C**

**A**

**B**

**Enriched**

**Enriched**

**Enriched**

**Restricted**

**Restricted**

**Restricted**

**Restricted**

**Enriched**

**C**

**B**

Graph A shows the faecal microbiota (*n* = 31) on day 42, with taxa with a mean abundance of < 1.0% grouped together. Graph B shows the ileal microbiota (*n* = 31) on day 90, with taxa with a mean abundance of < 0.5% grouped together, while graph C shows the faecal microbiota (*n* = 28) on day 90, with taxa with a mean abundance of < 1.0% grouped together. For each graph, the colour legend is organised in descending order of mean abundance.


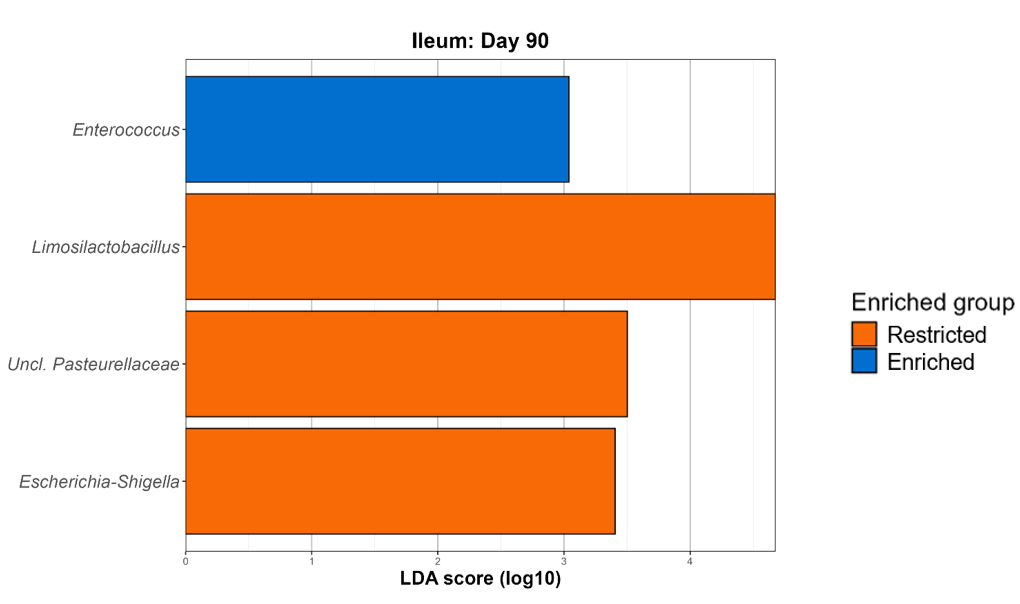


**Figure S5. Genera enriched among the restricted and enriched groups on day 90 (ileum) as identified by LEfSe analysis.**

For each genus deemed significantly different between the two groups, the LDA score is visualised on the $x$-axis, with a threshold of > 3 chosen for a significant difference.


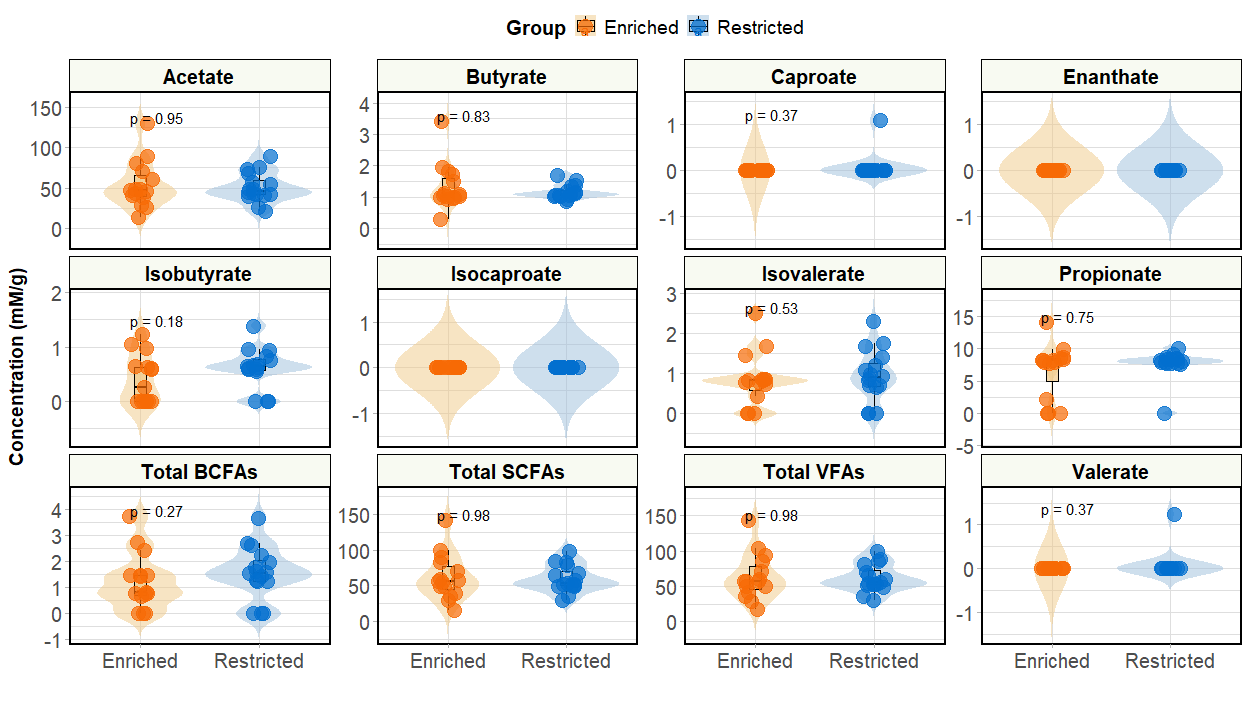


**Figure S6. Ileal VFA concentrations on day 90.**

The violin plots display VFA concentrations from acetate to enanthate, with total SCFAs, BCFAs, and VFAs also shown. Compromised pigs are represented in orange, and optimal in blue. *P*-values, as calculated by the Wilcoxon test, are shown.

**
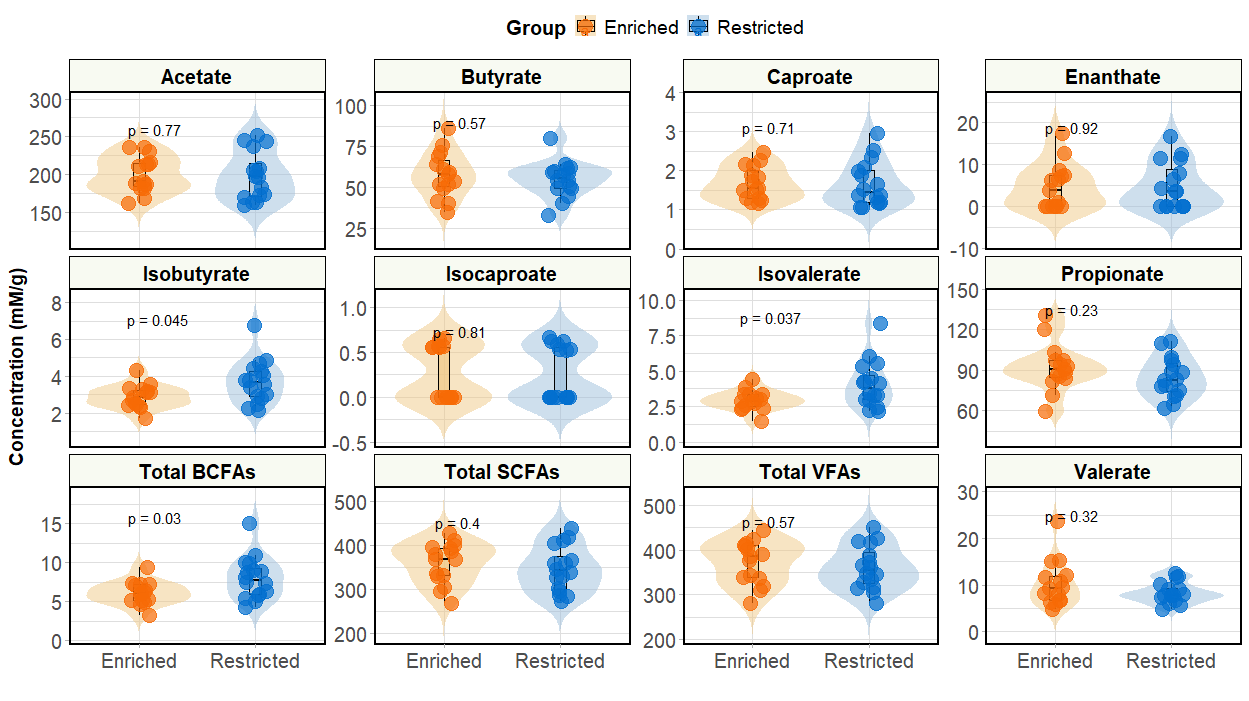
**

**Figure S7. Colonic VFA concentrations on day 90.**

The violin plots display VFA concentrations from acetate to enanthate, with total SCFAs, BCFAs, and VFAs also shown. Compromised pigs are represented in orange, and optimal in blue. *P*-values, as calculated by the Wilcoxon test, are shown.


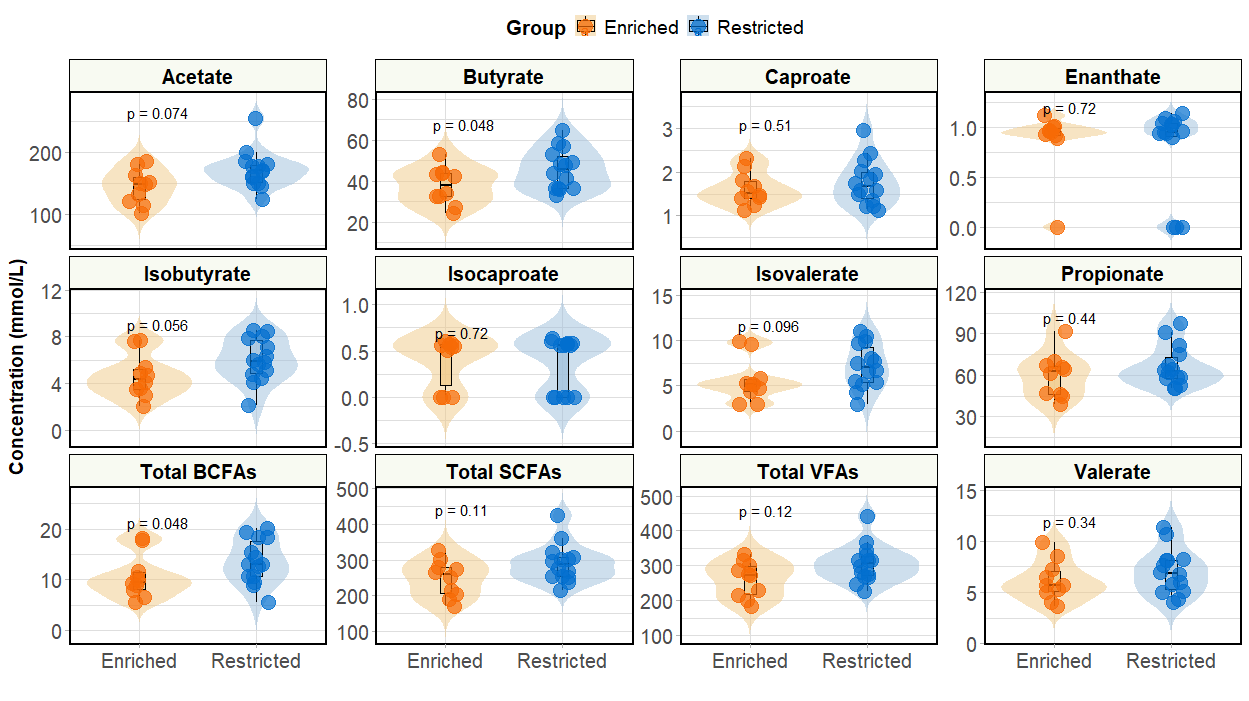


**Figure S8. Faecal VFA concentrations on day 90.**

The violin plots display VFA concentrations from acetate to enanthate, with total SCFAs, BCFAs, and VFAs also shown. Compromised pigs are represented in orange, and optimal in blue. *P*-values, as calculated by the Wilcoxon test, are shown.
